# Cell membrane coating integrity affects the internalization mechanism of biomimetic nanoparticles

**DOI:** 10.1038/s41467-021-26052-x

**Published:** 2021-09-30

**Authors:** Lizhi Liu, Xuan Bai, Maria-Viola Martikainen, Anna Kårlund, Marjut Roponen, Wujun Xu, Guoqing Hu, Ennio Tasciotti, Vesa-Pekka Lehto

**Affiliations:** 1 Department of Applied Physics, University of Eastern Finland, 70210 Kuopio, Finland; 2 Department of Engineering Mechanics, State Key Laboratory of Fluid Power and Mechatronic Systems, Zhejiang University, 310027 Hangzhou, China; 3 Department of Environmental and Biological Sciences, University of Eastern Finland, 70210 Kuopio, Finland; 4 Institute of Public Health and Clinical Nutrition, University of Eastern Finland, 70211 Kuopio, Finland; 5 IRCCS San Raffaele Pisana Hospital and San Raffaele University, Rome, Italy; 6 Sclavo Pharma, Siena, Italy

**Keywords:** Bioinspired materials, Nanoparticles, Biomedical engineering, Nanotechnology in cancer

## Abstract

Cell membrane coated nanoparticles (NPs) have recently been recognized as attractive nanomedical tools because of their unique properties such as immune escape, long blood circulation time, specific molecular recognition and cell targeting. However, the integrity of the cell membrane coating on NPs, a key metrics related to the quality of these biomimetic-systems and their resulting biomedical function, has remained largely unexplored. Here, we report a fluorescence quenching assay to probe the integrity of cell membrane coating. In contradiction to the common assumption of perfect coating, we uncover that up to 90% of the biomimetic NPs are only partially coated. Using in vitro homologous targeting studies, we demonstrate that partially coated NPs could still be internalized by the target cells. By combining molecular simulations with experimental analysis, we further identify an endocytic entry mechanism for these NPs. We unravel that NPs with a high coating degree (≥50%) enter the cells individually, whereas the NPs with a low coating degree (<50%) need to aggregate together before internalization. This quantitative method and the fundamental understanding of how cell membrane coated NPs enter the cells will enhance the rational designing of biomimetic nanosystems and pave the way for more effective cancer nanomedicine.

## Abstract (web summary)

Cell membrane coating of nanomaterials has become an attractive method of improving targeting, residence and biocompatibility. Here, the authors demonstrated that most nanoparticles are only partially coated by standard methods, and show the coating degree can impact the biological fate of nanoparticles.

## Introduction

Cell membrane coating has been introduced as an advanced process that adopts a simple top-down approach for functionalizing synthetic nanoparticles (NPs) with the complex functionalities associated with natural cell membranes^1^. Specifically, cell membrane-coated NPs inherently mimic the surface properties of the source cells and thus acquire many unique characteristics, such as superior biocompatibility, decreased uptake by macrophage cells, prolonged circulation lifetimes, and enhanced tumor penetration^2–5^. Based on these advantages, a wide variety of cell types including red blood cells (RBCs), platelets, white blood cells, cancer cells, stem cells and even bacteria, have been employed as sources of cell membranes to coat synthetic NPs^6,7^. Among their applications, just to name a few: red blood cell membrane-cloaked magnetic mesoporous silica NPs for cancer therapy^8^, platelet‐camouflaged poly(lactic-co-glycolic acid) (PLGA) NPs for targeting and detection of atherosclerosis^9^, neutrophil membrane-coated polymeric NPs capable of evoking anti-inflammation^10^, and cancer cell membrane-coated hybrid NPs for tumor fluorescence imaging^11^. It is a general assumption inherent in most of these coating approaches that the cell membranes uniformly cover the entirety of the NPs surface, thus forming an integrated core-shell structure. However, this top-down biomimetic procedure first requires to disrupt the cell integrity to obtain cell membranes, and then to fuse them with the core NPs by applying external forces (such as extrusion or sonication). We hypothesized that these synthetic methods could result in a lack of full integrity of the re-assembled cell membrane coatings. If one considers biomimetic NPs, then the loss of lipid shell integrity could affect their biomedical functionalities, such as cargo leakage in drug delivery systems^12^, undesired biomolecules adsorption occurred in physiological fluids^13,14^, changes in the NPs’ mechanical properties and last but not least, alterations in the molecular affinity of the membranes^15^. Therefore, in addition to the reserved membrane proteins, it is essential to investigate whether the integrity of the cell membrane can be replicated onto the biomimetic NPs.

Existing methods for confirming a successful cell membrane coating have largely relied on transmission electron microscope (TEM) observation, dynamic light scattering (DLS) measurement, evaluation of zeta potential, colloidal stability test in phosphate-buffered saline (PBS) or fetal bovine serum (FBS), and sodium dodecyl sulfate-polyacrylamide gel electrophoresis (SDS-PAGE) analysis^16–21^. However, these are qualitative methods that they fail to evaluate the degree and variability of the coating in a statistically meaningful manner. Herein, first we develop a fluorescence quenching assay to calculate the percentage of fully coated NPs. We then apply this method to test the ratio of full coating of cell membrane-coated NPs prepared by different synthetic methods (extrusion and sonication), using core NPs with variable sizes, charges and structures, and multiple cell membrane sources (RBC, platelet, cancer cell and macrophage). We find that the ratio of full coating under different conditions never exceeds 20%, indicating that the great majority of the biomimetic NPs are only partially coated. In addition, we demonstrate that despite of this partial coating, biomimetic NPs still exhibit source cell-specific targeting abilities, owing to the cell adhesion molecules present in the source cells. Finally, we systematically investigate the mechanisms underlying the coating degree-dependent NP-cell interactions to provide a framework for understanding the internalization of cell membrane-coated NPs.

## Results

### Quantification of ratio of full cell membrane coating

**Fig. 1:**
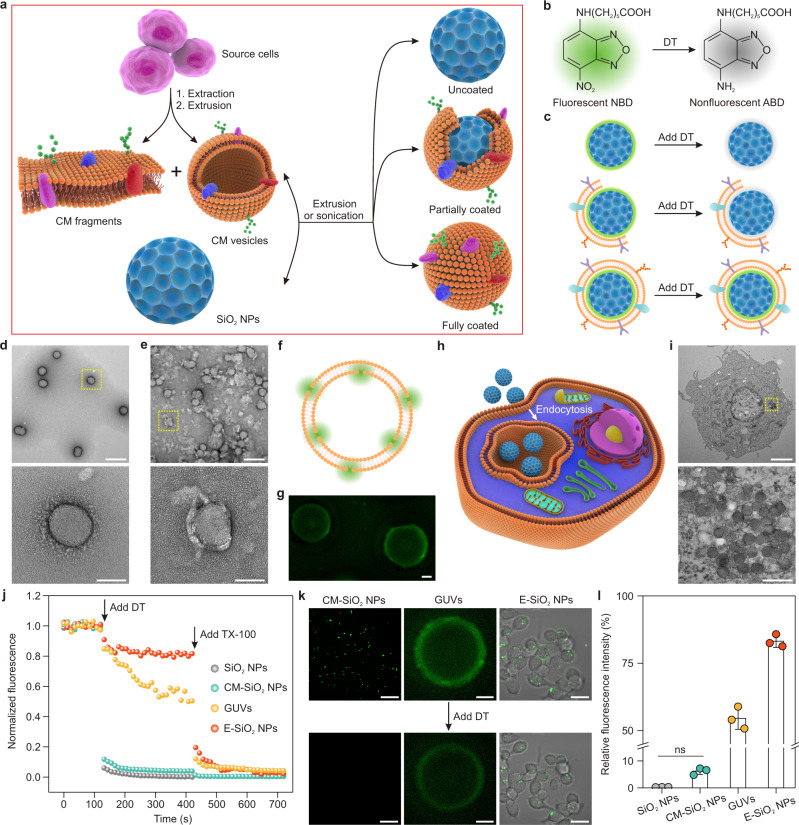
Quantification of ratio of full cell membrane coating. **a** Schematic illustration of the preparation procedure of the cell membrane-coated mesoporous SiO_2_ nanoparticles (CM-SiO_2_ NPs). Membrane materials including fragments and vesicles were derived from source cells through extraction and extrusion processes. Then, the membrane materials were further fused with SiO_2_ NPs to obtain CM-SiO_2_ NPs by extrusion or sonication. The resulting mixture contained uncoated SiO_2_ NPs, partially coated SiO_2_ NPs and fully coated SiO_2_ NPs. **b** Reduction of the fluorescent nitro-2,1,3-benzoxadiazol-4-yl (NBD) to the nonfluorescent 7-amino-2,1,3-benzoxadiazol (ABD) with dithionite (DT). **c** Schematic representation of probing the integrity of the cell membrane coating by the fluorescence quenching assay. In the presence of DT, which is a membrane-impermeant fluorescence quencher, the fluorescence of uncoated SiO_2_ NPs (labeled with NBD) and partly coated SiO_2_ NPs disappeared while only that of fully coated SiO_2_ NPs was retained. **d**, **e** TEM images of bare SiO_2_ NPs (**d**) and CM-SiO_2_ NPs (**e**). Insets are magnified images of the areas highlighted with the respective yellow dashed box. Scale bars, 200 nm (top); 50 nm (bottom). **f** Schematic representation of symmetrically distributed NBD-labeled giant unilamellar vesicles (GUVs). **g** Typical confocal laser scanning microscopy (CLSM) image of symmetrical GUVs. Scale bar, 20 μm. **h** Schematic showing that SiO_2_ NPs were endocytosed into CT26 cells after incubation 24 h, then localized within endosomes. **i** TEM images of CT26 cell endocytosed SiO_2_ (E-SiO_2_) NPs. Inset shows a magnified image of the area highlighted with a yellow dashed box. Scale bars, 2 μm (top); 200 nm (bottom). **j** Representative fluorescence traces corresponding to DT treatment of SiO_2_ NPs, CM-SiO_2_ NPs, GUVs and E-SiO_2_ NPs. DT was first added at *t* = 120 s to bleach the fluorescence of NPs with a defective cell membrane coating. After a stable baseline was obtained, 1% Triton X-100 (TX-100) was added at 420 s to disrupt the integrated cell membrane and bleach the remaining fluorescence of fully coated NPs. **k** CLSM images of CM-SiO_2_ NPs, GUVs and E-SiO_2_ NPs before (top) and after (bottom) addition of DT. Scale bars, 20 μm. **l** Quantification of the relative fluorescence intensity of CM-SiO_2_ NPs as well as the blank control (bare SiO_2_ NPs) and positive control (GUVs and E-SiO_2_ NPs). Data represents mean ± SD (*n* = 3). One-way ANOVA followed by post hoc Tukey test was used to determine the significance of data. ns: not significant.

The acquisition of cell membrane material used for coating usually involves two steps: extracting cell membranes from harvested cells and extrusion through porous membranes to create cell membrane-derived vesicles^6^. We hypothesized that this resulting mixture of cell membrane fragments and vesicles (Supplementary Fig. 1) fusing with NPs by extrusion or sonication could result in three classes of products characterized by the degree of membrane integrity: uncoated, partially coated and fully coated NPs (Fig.1a). In order to probe the integrity of the cell membrane coating, we developed a fluorescence quenching assay in which the NPs labeled with fluorescent nitro-2,1,3-benzoxadiazol-4-yl (NBD) by sequential chemical covalent coupling (Supplementary Fig. 2) were treated with dithionite (DT), a negatively charged reducing regent that cannot cross membranes^22,23^. DT reduces NBD to 7-amino-2,1,3-benzoxadiazol (ABD), which irreversibly quenches the fluorescence of exposed NBD-labeled NPs (Fig. 1b). We hypothesized that if the NPs were fully coated, then the fluorescence signal would remain after the addition of the DT quencher into the solution. In contrast, if the NPs were only partially coated or totally uncoated, the fluorescence intensity would progressively disappear due to the reduction of the NBD dye with DT (Fig. 1c). Therefore, by measuring the remaining fluorescence, we would be able to calculate the proportion of fully coated NPs.

To investigate the feasibility of this approach, mouse colon carcinoma (CT26) cells were used as a model cancer cell line from which cell membranes were extracted, while ~70 nm mesoporous SiO_2_ NPs (Supplementary Fig. 3) were selected as a model core nanomaterial due to their excellent drug delivery and imaging capabilities^24^. The purified cancer cell membranes were first obtained by emptying cells of their intracellular components by applying a combination of hypotonic lysing, mechanical membrane disruption, and differential centrifugation^25^. The cell membrane material used for coating was then formed by physical extrusion through a 400 nm porous polycarbonate membrane. Finally, the resulting cell membrane materials were coextruded with SiO_2_ NPs through a 200 nm porous polycarbonate membrane to obtain cell membrane-coated SiO_2_ (CM-SiO_2_) NPs. The successful fusion of cell membrane materials with SiO_2_ NPs was directly visualized by transmission electron microscopy (TEM), which clearly showed the spherical SiO_2_ core surrounded by an outer lipid bilayer shell of around 9 nm in thickness (Fig. 1d, e). These results were comparable to the reported thickness of natural cellular membrane phospholipid bilayers (5–10 nm)^26,27^. Furthermore, dynamic light scattering (DLS) measurements revealed that the hydrodynamic diameters of SiO_2_ NPs slightly increased from 118.6 ± 0.4 nm to 139.8 ± 5.4 nm after cell membrane coating (Supplementary Fig. 4a), confirming the presence of the coating cell membrane around the NPs. Meanwhile, the surface zeta potential changed from −41.2 ± 0.9 mV to −36.0 ± 0.3 mV (Supplementary Fig. 4b), due to charge screening by the cell membrane. To further verify the successful cell membrane coating on SiO_2_ NPs, CT26 cells were incubated with CM-SiO_2_ NPs for 4 h, where SiO_2_ NPs and cell membrane were labeled with Cyanine 5 (Cy5) and 1,1′-dioctadecyl-3,3,3′,3′-tetramethylindocarbocyanine perchlorate (DiI), respectively. Confocal laser scanning microscopy (CLSM) images demonstrated that the red fluorescence derived from SiO_2_ NPs matched well with the green fluorescence derived from CT26 cell membrane (Supplementary Fig. 5), indicating the successful formation of CM-SiO_2_ NPs. Furthermore, sodium dodecyl sulfate-polyacrylamide gel electrophoresis (SDS-PAGE) analysis demonstrated that the protein composition of CM-SiO_2_ NPs was similar to that of cell membranes (Supplementary Fig. 4c), indicative of a good retention of the characteristic proteins presents in the membrane of these cancer cells. Importantly, the obtained CM-SiO_2_ NPs displayed good colloidal stability after 6 days of storage in phosphate-buffered saline (PBS) at a concentration of 1 mg/mL (Supplementary Fig. 4d). Collectively, these results confirmed the presence of cancer cell membranes on SiO_2_ NPs cores, while no general conclusion could be drawn on the extent of the membrane coating.

In addition to the CM-SiO_2_ NPs, we prepared NBD-labeled giant unilamellar vesicles (GUVs; Fig. 1f, g) via a natural swelling method^28^ and endocytosed SiO_2_ (E-SiO_2_) NPs (Fig. 1h) as the control groups to test whether DT is able to cross the cell membrane. In order to produce E-SiO_2_ NPs that were encapsulated by the membrane structures of living cells, CT26 cells were incubated with SiO_2_ NPs (200 μg/mL) for 24 h. TEM images revealed that E-SiO_2_ NPs were mainly located in the lysosomes (Fig. 1i), indicating that the SiO_2_ NPs were fully surrounded by native cellular membranes. With the CM-SiO_2_ NPs, GUVs and E-SiO_2_ NPs in hand, we next tested the proposed fluorescence quenching assay. After addition of DT to the sample (Fig. 1j), the fluorescence of bare SiO_2_ NPs decreased immediately and was completely lost after 5 min, consistent with a reduction of the free NBD dye. With respect to the NBD-labeled GUVs, we observed a rapid decrease in fluorescence (approximately 45%), consistent with the assumption that the only outer leaflet NBD-phospholipids were reduced, while the inner-leaflet phospholipids were protected. Following the addition of Triton X-100 (TX-100), the fluorescence intensity was further reduced by ~50%, as TX-100 can disrupt the GUVs and allow DT to react with the NBD-labeled phospholipids in the inner leaflet. Notably, a rapid decrease (~90%) in the fluorescence of the CM-SiO_2_ NPs was observed after the addition of DT, indicating that the partial coating was dominant. To rule out the possibility that this greater fluorescence reduction could be due to protein-mediated permeation of DT across the membrane rather than partial coating, we measured the fluorescence of E-SiO_2_ NPs, which were protected by integrated living cellular membranes. As expected, only ~20% of the fluorescence was lost upon addition of the DT, which could be attributed to the free SiO_2_ NPs adsorbed onto the surface of cellular membranes. Similar to GUVs, a complete reduction was seen after permeabilizing the cells with TX-100, indicating that the remaining fluorescence of E-SiO_2_ NPs had resulted from the protection of cellular membranes. This fluorescence quenching was further visualized in the CLSM images, where the fluorescence signal of CM-SiO_2_ NPs completely disappeared, while that of GUVs and E-SiO_2_ NPs remained after the addition of DT (Fig. 1k). We attributed the remaining fluorescence of GUVs to the NBD inserted in the inner leaflet. These results confirmed that DT was not able to cross the cell membrane, unless the cell membranes were not fully integrated, making the NBD-labeled SiO_2_ NPs available for reaction. In addition, we observed that the adsorption of bovine serum albumin (BSA) on NPs didn’t reduce the quenching (Supplementary Fig. 6), indicating that the protein adsorption has little effect on our proposed fluorescence quenching assay. The quantification analysis revealed that the ratio of full coating of CM-SiO_2_ NPs was only ~6% (Fig. 1l), which was due to the existence of a few fully coated SiO_2_ NPs (Supplementary Fig. 7). Altogether, these results demonstrated that most of SiO_2_ NPs were partially coated in contradiction to the common assumption that the extrusion process results in fully coated NPs^20,29,30^.

### Validation of partial cell membrane coating

**Fig. 2:**
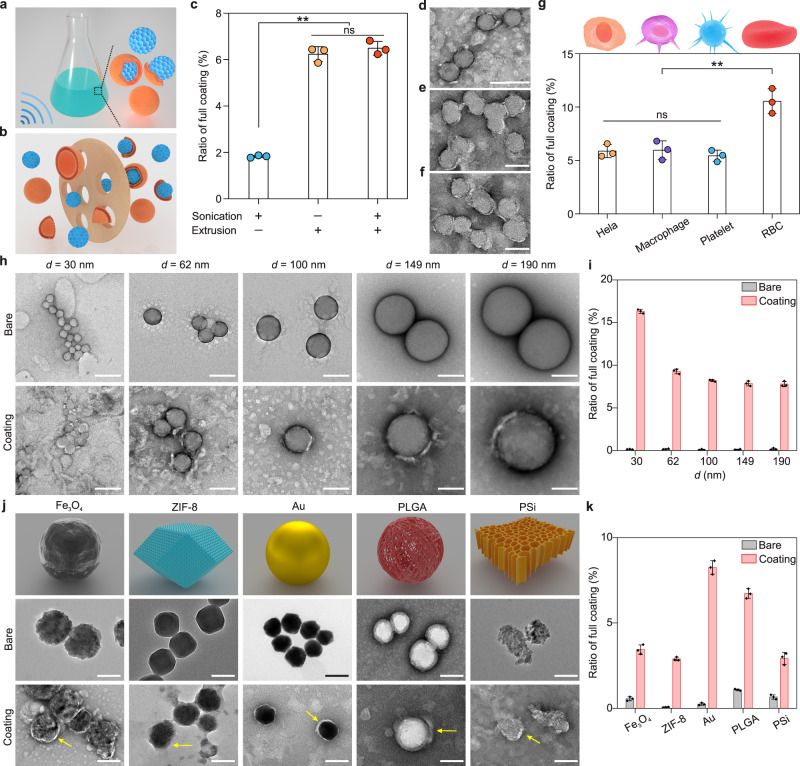
Validation of partial cell membrane coating under different experimental designs. **a**, **b** Schematic illustration of the preparation of cell membrane-coated NPs with a sonication method (**a**) and a physical co-extrusion method (**b**). **c** Quantification of the ratio of full cell membrane coating with different coating methods (sonication, extrusion, and combined sonication-extrusion). **d−f** TEM images of CM-SiO_2_ NPs fabricated using sonication (**d**), extrusion (**e**), and combined sonication-extrusion (**f**). Scale bars, 100 nm. **g** Quantification of the ratio of full cell membrane coating for SiO_2_ NPs coated with different source cell membrane materials (HeLa, macrophage, platelet, and RBC). **h** TEM images of different sizes of nonporous Stöber SiO_2_ NPs before and after coating with cell membranes. Scale bars, 100 nm. **i** Quantification of the ratio of full cell membrane coating for cell membrane-coated nonporous Stöber SiO_2_ NPs of different sizes. **j** TEM images of Fe_3_O_4_ NPs, ZIF-8 NPs, Au NPs, PLGA NPs, and porous silicon (PSi) NPs before and after coating with cell membranes. Scale bars, 100 nm. **k** Quantification of the ratio of full cell membrane coating for different core materials (Fe_3_O_4_ NPs, ZIF-8 NPs, Au NPs, PLGA NPs, and PSi NPs). Data represents mean ± SD (*n* = 3). One-way ANOVA followed by post hoc Tukey test was used to determine the significance in **c** and **g**. *p* = 1.7E-6 (**c**: sonication+ and extrusion– vs. sonication– and extrusion+), *p* = 1.2E-6 (**c**: sonication+ and extrusion– vs. sonication+ and extrusion+), *p* = 6.1E-4 (**g**: RBC vs. Hela), *p* = 7.0E-4 (**g**: RBC vs. macrophage), *p* = 3.3E-4 (**g**: RBC vs. platelet). **p < 0.01. ns: not significant.

Next, we applied the proposed quantification method to compare the cell membrane integrity of different coating approaches. To date, ultrasonic fusion (Fig. 2a) and membrane extrusion (Fig. 2b) have been the two most commonly used methods in the fabrication of cell membrane-coated NPs. Among them, the mechanical force imposed by ultrasonic energy or mechanical extrusion leads to a disruption of the membrane’s structure, followed by the spontaneous formation of the cell membrane coating^1^. To explore the effect of coating methods on membrane integrity, we compared the ratio of full cell membrane coating of three methods: sonication, extrusion and a combination of these two approaches (Fig. 2c–f). Although we found the ratio of full coating was low with all the three methods (sonication: 1.8 ± 0.1%; extrusion: 6.2 ± 0.3%; combined sonication-extrusion: 6.5 ± 0.3%), it seemed that the extrusion process was more efficacious than sonication in the formation of a full cell membrane coating. Given that these top-down biomimicry approaches require that cell membranes and NPs come together under conditions of strong mechanical stress, we further investigated whether using milder coating approaches (e.g., simple incubation with cells) could retain the membrane integrity of biomimetic NPs. To do so, the exosome membrane-coated NPs that were recovered after exocytosis of the NPs previously endocytosed by cancer cells^31^ were chosen as an example of a milder coating method. After the CT26 cells were incubated with positively charged mesoporous SiO_2_ NPs, we first collected the exocytosed SiO_2_ (Ex-SiO_2_) NPs by centrifugation of the supernatants (Supplementary Fig. 8a). Upon membrane coating, an increase in the hydrodynamic diameter from 132.8 ± 5.8 nm to 162.8 ± 4.4 nm was detected (Supplementary Fig. 8b) and the zeta potential of SiO_2_ NPs changed from +26.2 mV to −23.7 mV (Supplementary Fig. 8c), validating the successful preparation of Ex-SiO_2_ NPs. TEM imaging further confirmed the presence of membrane structures on the SiO_2_ NPs surfaces (Supplementary Fig. 8d-f). Consistent with the top-down approach, the ratio of full coating of Ex-SiO_2_ NPs was approximately 6% which was comparable with the value of the CM-SiO_2_ NPs (Supplementary Fig. 8g), suggesting that this natural coating method was also unable to generate a complete membrane integrity for the resultant biomimetic NPs.

Since the cell membranes extracted from different cells contain a unique set of surface proteins, a variety of cell types were used as a source of membrane coating material to confer different functionalities onto the NPs. To examine whether the source of cell membrane materials could affect the cell membrane integrity of the resultant biomimetic NPs, membranes derived from four different cell types including cancer cells (HeLa), immune cells (RAW264.7 macrophages), platelets and RBCs, were used to construct biomimetic NPs. A partial coating was identified also when mesoporous SiO_2_ NPs were coated with these different cell membranes (Fig. 2g), consistently with what observed with the CT26 cell membranes. We noted that RBC-based NPs did exhibit a higher ratio of full coating (~10.5%) than membranes obtained from other cells, probably because of their well-preserved cell membrane structure after hypotonic lysis and extrusion (Supplementary Fig. 9). It has been recently reported that the surface charge of NPs plays a key role in the formation of supported lipid bilayers on NPs^32^. Inspired by this, we further analyzed the cell membrane integrity of positively and negatively charged NPs (Supplementary Fig. 10a–d), in which positively charged NPs were prepared without using succinic anhydride modification (Supplementary Fig. 2). Compared with the negatively charged NPs that can be extruded easily, the mixture of the positively charged NPs and negatively charged membranes resulted in extensive aggregation (Supplementary Fig. 10a) through the strong electrostatic interactions that impede the extrusion process. This aggregation caused a significant decrease in ratio of full coating (Supplementary Fig. 10e), indicating that the presence of a negative surface charge favored the formation of a full cell membrane coating, consistent with its reported roles in coating^33^. In addition, the strong affinity between positively charged NPs and negatively charged cell membranes could cause the collapse of the fluidic lipid bilayer, resulting in the arrangement failure of the local lipid upon the full coverage process^34^. Furthermore, to ascertain whether higher amounts of cell membrane materials could result in higher rates of fully coated NPs, we measured the ratio of full coating by increasing the weight ratio of the cell membrane materials to NPs from 0 to 5 (Supplementary Fig. 10f). As expected, increasing the cell membrane content, gradually improved the ratio of full coating, which reached a maximum value (~16.6%) at a weight ratio of 5. However, further increases in the cell membrane content caused a significant blocking of the porous membrane that impeded the extrusion process.

On the basis of the knowledge that the formation of a supported lipid bilayer on NPs can be influenced by the curvature of NPs^35^, we hypothesized that the size of core materials would also be an important parameter determining the integrity of the cell membrane of our biomimetic NPs. To test this hypothesis, we first prepared a series of nonporous Stöber SiO_2_ NPs with different diameters, which were achieved by tuning the amounts of ammonium hydroxide in the synthetic procedure (Supplementary Table 1). The TEM images and size distribution histograms in Supplementary Fig. 11a–f show that the nonporous Stöber SiO_2_ NPs had average sizes of 30 ± 2.9, 62 ± 8.1, 100 ± 7.0, 149 ± 7.8, and 190 ± 8.6 nm. To obtain cell membrane-coated NPs, each of these nonporous Stöber SiO_2_ NPs was extruded with an equal mass of CT26 cell membrane vesicles through polycarbonate membranes with appropriate pore sizes (200 nm for 30 and 63 nm NPs, and 400 nm for 100, 149, and 190 nm NPs). DLS measurements revealed that after cell membrane coating, the diameter of these differently sized NPs was increased by 10–20 nm, whereas the zeta potential was decreased (Supplementary Fig. 11g, h). Consistent with the results discussed above, a partial coating was mostly observed in these differently sized NPs, even in the case of the smallest NPs with a diameter of 30 nm (Fig. 2h), which was further confirmed by the ratio of full coating results (Fig. 2i). Furthermore, we noted that the ratio of full coating of the smallest NPs (~15%) was much higher than that of the other NPs, which could be attributed to its favorable bending energy requiring a lower interaction force to pull the membrane to the surface^35–37^.

According to what needs to be ultimately delivered to the targeted cells or tissues, the synthetic core materials are also important in the design of an effective cell membrane‐coated nano-construct. Hence, we next focused on understanding the effect of different core materials on the integrity of the membrane coating. To this end, we selected NPs made of magnetite (Fe_3_O_4_), zeolitic imidazole framework-8 (ZIF-8), gold (Au), PLGA, and porous silicon (PSi). Coating of CT26 cell membranes with these different NPs led to a consistent increase of the hydrodynamic size (Supplementary Fig. 12a) and of a change in the zeta potential (Supplementary Fig. 12b), demonstrating the presence of membrane coatings on the surfaces of these NPs. The partial coating of these NPs was clearly observed in the TEM images (Fig. 2j), and this was further supported by the ratio of full coating results (Fig. 2k). We attributed the highest ratio of full coating observed in Au NPs (8.3%) to the strong affinity of the free sulfhydryl group on the membrane protein for the Au NPs, which provides an extra force for cell membrane coating. Collectively, the experiments described above indicate that the partial coating is a general phenomenon existing in cell membrane‐based biomimetic NPs.

### Cellular uptake of partially coated NPs

**Fig. 3:**
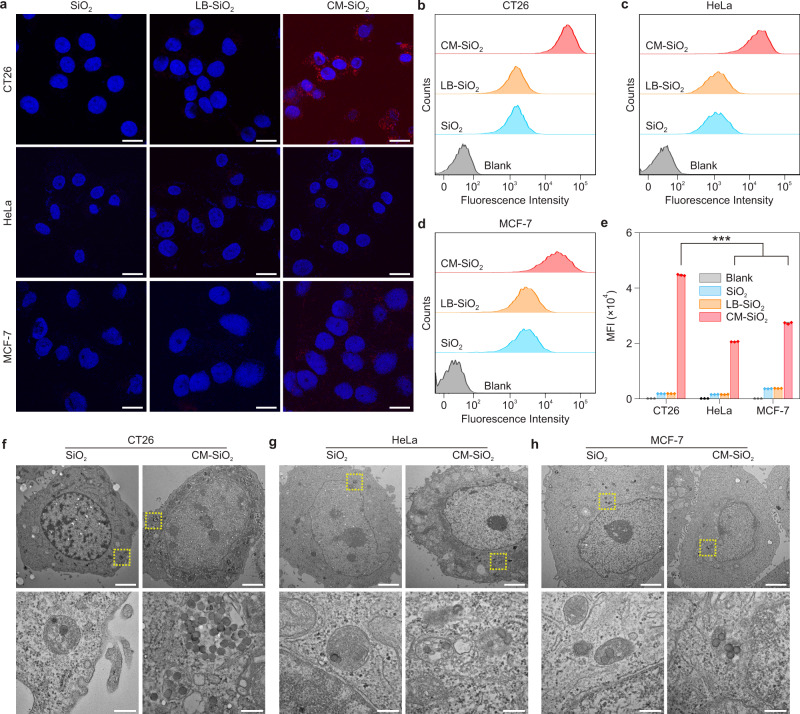
In vitro characterization of the homologous targeting capabilities of CM-SiO_2_ NPs. **a** Representative CLSM images of three cell lines (CT26, HeLa and MCF-7) after 4 h incubation with SiO_2_ NPs, LB-SiO_2_ NPs and CM-SiO_2_ NPs. The SiO_2_ cores were labeled with Cy5 (red), and the cell nuclei were stained with 4’,6-diamidino-2-phenylindole (DAPI; blue). Scale bars, 20 μm. **b**–**d** Flow cytometric analysis of CT26 cells (**b**), HeLa cells (**c**) and MCF-7 cells (**d**) incubated with blank solution, SiO_2_ NPs, LB-SiO_2_ NPs and CM-SiO_2_ NPs. **e** Quantification of the mean fluorescence intensities for the three cell lines (CT26, HeLa, and MCF-7). Data represents mean ± SD (*n* = 3). One-way ANOVA followed by post hoc Tukey test was used to determine the significance of data. ***p < 0.001. **f**–**h** TEM images of CT26 cells (**f**), HeLa cells (**g**), and MCF-7 cells (**h**) after 4 h incubation with SiO_2_ NPs and CM-SiO_2_ NPs. Insets below are magnified of each image in the area highlighted with the respective yellow dashed box. Scale bars, 2 μm (top); 200 nm (bottom).

To explore whether the partially coated CM-SiO_2_ NPs displayed self-recognition of the corresponding homologous cell line and enhanced cellular internalization, we checked the NP uptake capability of three cell lines including CT26, HeLa and MCF-7 (Fig. 3). The biocompatibility of CM-SiO_2_ NPs was tested on CT26 cells and showed negligible deleterious effects (Supplementary Fig. 13), in line with previous reports of membrane-coated NPs^38^. The CLSM images showed that CT26 cells exhibited a higher red fluorescence (Cy5-labeled NPs-based formulations) as compared to the other cell lines after incubation with CM-SiO_2_ NPs, whereas all of the cell lines treated with SiO_2_ NPs and DOPC lipid bilayer coated SiO_2_ (LB-SiO_2_) NPs displayed a similar weak red fluorescence (Fig. 3a), verifying the targeting ability of the cell membrane to its source tumor cell. We measured quantitatively the intracellular uptake of the different cell types by using flow cytometry (Fig. 3b–e). It demonstrated that after 4 h incubation with NPs, the fluorescence intensity of homologous CT26 cells was nearly 2.2- and 1.6-fold higher than that of HeLa cells and MCF-7 cells, respectively, indicating the specific binding ability of CM-SiO_2_ NPs to the homologous CT26 cells. This source cell-specific targeting property of CM-SiO_2_ NPs was further evidenced by TEM in CT26, HeLa and MCF-7 cells (Fig. 3f–h and Supplementary Fig. 14), in which a large number of CM-SiO_2_ NPs localized in the endocytic vesicles could be observed in CT26 cells while CM-SiO_2_ NPs were rarely present in other cell lines. Taken together, our data suggested that even a partial coating of SiO_2_ NPs with CT26 cells membrane was sufficient to increase the affinity of the particles to the source cancer cells, a functionality that can be attributed to the transference of cell adhesion molecules with homotypic binding properties^38^.

We next probed whether the coating of the cancer cell membrane also affected the internalization of the NPs by macrophages. In contrast to the case of tumor cells, the results of CLSM (Supplementary Fig. 15a) and flow cytometry (Supplementary Fig. 15b, c) both indicated that the macrophage cell line RAW264.7 showed significantly reduced binding and/or internalization of CM-SiO_2_ NPs as compared to that of bare SiO_2_ NPs and LB-SiO_2_ NPs. This was in a good agreement with the results from a previous study showing that the presence of a cell membrane coating reduced the clearance of NPs by the reticular endothelial system^39,40^, suggesting that even partial cell membrane decoration seemed to suffice in improving the short half-life of traditional NPs.

### Endocytic entry mechanism study of partially coated NPs

Our in vitro targeting results raised a fundamental question regarding how partially coated NPs enter the cells. To systematically address this issue, we first sought to find out the endocytic pathway of partially coated NPs by CT26 cells. To do so, we pre-treated cells with a low temperature (4 °C) and various pharmacological endocytosis inhibitors before the addition of CM-SiO_2_ NPs (Supplementary Fig. 16). The uptake of CM-SiO_2_ NPs was notably inhibited at 4 °C, suggesting they were being internalized via an energy-dependent endocytosis process. The cellular uptake of CM-SiO_2_ NPs was reduced to ~14% in the presence of chlorpromazine (CPZ; inhibitor of clathrin-dependent endocytosis), whereas exposure to genistein (GEN; inhibitor of caveolin-dependent endocytosis) and cytochalasin D (CytD; inhibitor of macropinocytosis) had negligible effects, indicating that partially coated NPs were internalized primarily by clathrin-dependent endocytosis, generally referred to as “receptor-mediated endocytosis”^41^.

**Fig. 4:**
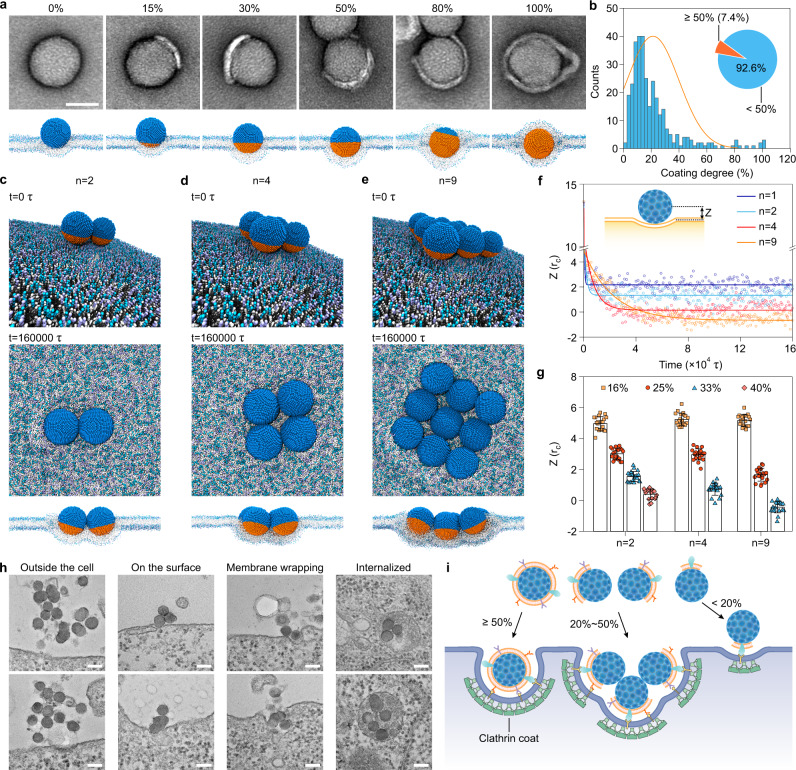
Endocytic entry mechanism of partially coated NPs. **a** Top: TEM images of SiO_2_ NPs with different cell membrane coating degrees (0%, 15%, 30%, 50%, 80%, and 100%). Scale bar, 50 nm. Bottom: the corresponding final dissipative particle dynamics (DPD) simulation snapshots of the wrapping of the CM-SiO_2_ NPs by a modeled cell membrane. It shows that the wrapping of a single CM-SiO_2_ NP with a higher coating degree is easier than that of an NP with a lower coating degree. **b** Cell membrane coating degree distribution of as-prepared CM-SiO_2_ NPs, which is calculated from TEM images (*n* = 325). The inset shows the proportion of SiO_2_ NPs with a low cell membrane coating degree (<50%). **c**–**e** Typical DPD simulation snapshots of multiple CM-SiO_2_ NPs: aggregation number (*n*) = 2 (**c**), 4 (**d**), and 9 (**e**). The coating degree of each NP is 33%. The top panel (*t* = 0 τ) shows the setup of the simulation system and the other panels (*t* = 160,000 τ) displays the final equilibrated NP-membrane structure at the top view and the profile view. **f** Time evolution of CM-SiO_2_ NPs positions along the membrane normal direction. *Z* represents the distance between the center of the NP and the cellular phospholipid bilayer (inset). **g** Comparison of positional change of CM-SiO_2_ NPs with different aggregated numbers (2, 4, and 9) and coating degrees (16%, 25%, 33%, and 40%). Data represents mean ± SD (*n* = 20). **h** TEM images showing the different states of CM-SiO_2_ NPs during receptor-mediated interactions with CT26 cells. Scale bars, 100 nm. **i** Schematic illustration of a possible endocytic entry mechanism for partially coated NPs.

In receptor-mediated endocytosis, the energy obtained upon NPs’ ligands binding to cell surface receptors requires an overcoming of the deformation energy of the membrane^42^. Given that the surface of CM-SiO_2_ NPs was partially covered with ligands, we next wondered whether such receptor–ligand binding strength would be strong enough to drive NPs over the energy barrier during the uptake. We did this by employing dissipative particle dynamics (DPD) simulations to simulate the receptor-mediated endocytosis of partially coated NPs (Supplementary Fig. 17; see more details in the section of Model and Simulation). Here, we defined the degree of cell membrane coating as the ratio of the cell membrane coating surface area to the total NP surface area (Supplementary Fig. 18). We first analyzed the effect of different coating degrees (0%, 15%, 30%, 50%, 80%, and 100%) on a single NP’s wrapping (Fig. 4a). Consistently, the previously reported TEM images of cell membrane-coated NPs supported the existence of partial coating^26,43,44^. This phenomenon could be attributed to the support effect that induced constraints on the lipid mobility^45^, as a non-constrained patch would adopt the thermodynamically favorable circular shape. The results revealed that a larger coating degree could induce more cell membrane bending around the CM-SiO_2_ NP, resulting in a lower z-position below the membrane and a higher wrapping ratio (Supplementary Fig. 19). It is noteworthy that during the clathrin-mediated endocytosis process, after entrapment by clathrin-coated pits, the NP could be completely internalized into the cell via active membrane shrinkage, a process that can be triggered by clathrin and actin filaments polymerization^46,47^. Based on this phenomenon, in our following simulations we chose a coating degree of 50% as the criteria to determine whether a single NP could be internalized by cells because its final position was close to the center of the phospholipid bilayer. However, the proportion of CM-SiO_2_ NPs with a coating degree larger than the critical coating degree was only 7.4% (Fig. 4b), suggesting that most of the NPs were unlikely to be individually internalized by the cells. This observation was further supported by examining the TEM images of the NPs internalization at different incubation time points (Supplementary Fig. 20). These observations showed that the CM-SiO_2_ NPs located in the early endosomes were mainly aggregated, while 9.5% NPs were individual in an early endosome. This value (9.5%) was comparable to the proportion (7.4%) of NPs with a coating degree greater than the critical coating degree, further supporting that the threshold value of the coating degree we selected was appropriate. Remarkably, the CT26 cells incubated with NPs at a lower concentration (1 μg/mL; Supplementary Fig. 21) still exhibited a similar ratio of individual NPs aggregated in a single early endosome to high concentration (50 μg/mL; Supplementary Fig. 20), suggesting that these NPs had to aggregate for the uptake.

Based on the above results of single NP simulation and TEM analysis, we hypothesized that the NPs with low coating degrees (<50%) aggregated together and provided more ligands to enter the cells. To test this hypothesis, we performed simulations examining the interaction between the cell membrane and the multiple CM-SiO_2_ NPs with a coating degree of 33% (aggregation number: 2, 4, and 9; Fig.4c–e). Interestingly, the NP aggregates were more likely to enter the cell membranes as the aggregation number increased (Fig. 4f). The final structure of the NP-membrane showed that the aggregated NPs rotate spontaneously after entering into the membranes to promote more ligands binding with the cell receptor (Supplementary Fig. 22a-c and Supplementary Movies 1–3). Regarding the aggregated NPs as an individual particle, such rotation provided more ligands on the side of the NP aggregates, which subsequently promoted the wrapping of the NPs (Supplementary Fig. 22c). Inspired by the simulation results, we further proposed a two-dimension geometric model to predict the required coating degree for half wrapping the NPs aggregates (Supplementary Fig. 23). Under these experimental conditions, the required coating degree for two NPs was about 29%−32% and that for three NPs was about 23%−26%, which was much smaller than the one required for internalizing an individual NP (50%). It should be clear that this predicted value would be slightly smaller than the actual three-dimension (n = 4, 9) required coating degree (possible reasons for this discrepancy are given in Supplementary Note 1).

Given that the coating degree of the CM-SiO_2_ NPs mostly varied from 5% to 50% (Fig. 4b), we explored the interactions between the membrane and the CM-SiO_2_ NPs with different coating degrees (16%, 25%, 33%, and 40%; Fig. 4g and Supplementary Fig. 24). With an increasing aggregation number, the NPs could more easily enter into the cells and a lesser coating degree was needed to achieve 50% wrapping by the membrane. However, it was found that when the coating degree decreased to 16%, the cooperation within the aggregated NPs was significantly weakened, resulting in a failure of the internalization by the cell membrane. This is because at such a low coating degree, the rotation cannot promote binding of any extra ligands to the receptors, which only occurs at much higher coating degrees (Supplementary Fig. 22d, e). Therefore, in the case of the NPs with a low coating degree (20%), increase in the aggregation number (n = 16) cannot solely promote their internalization (Supplementary Fig. 25). Remarkably, the aggregation number of NPs that enter the cells is limited by the size of clathrin-coated vesicles (approximately 100–300 nm)^48^. Taking into account the nonuniformity of the coating degrees within the NPs aggregates, the NPs with a very low coating degree (20%) may be able to enter the cell with the help of NPs with a higher coating degree (40%; Supplementary Fig. 26). Taken together, these results strongly indicate that the CM-SiO_2_ NPs can also enter into the cells by a process called aggregated cooperation when the coating degree is lower than 50%. To validate the prediction emerging from our computational model, we used TEM to capture the membrane-NPs structures at the different interaction stages (Fig. 4h), which demonstrated that the dispersed NPs would aggregate after adhering onto the membranes and subsequently first induce membrane wrapping and then internalization of the NP aggregates. Compared with the individually internalized NPs (Supplementary Fig. 27), multiple NPs are wrapped within a much larger endosome (200–400 nm) and thus need more proteins to actively bend the membrane.

Finally, putting the theoretical modeling and experimental results together, we proposed a possible endocytic entry mechanism for the partially coated NPs (Fig. 4i). The NPs with a high coating degree (≥50%) were able to undergo individual cell entry. By contrast, those NPs with a low coating degree (20%−50%) needed to first aggregate with each other on the cell’s surface before they could synergistically enter into cells. The aggregation number of NPs with low coating degrees highly depends on their degree of cell membrane coating. The NPs with a very low coating degree (<20%) may be hardly engulfed by the membranes even with cooperation with each other, but they are able to be engulfed when they can aggregate with NPs with higher coating degrees (e.g., ≥40%).

## Discussion

The main purpose of applying a cell membrane coating is to confer NPs with the complex functionalities necessary for effective biointerfacing (e.g., long circulation time and disease-relevant targeting)^3,49,50^. However, when we followed the standard protocols to prepare cell membrane-coated NPs, we demonstrated that more than 60% of the NPs possessed a coating degree below 20% (Fig. 4b). These NPs might encounter difficulties in entering the cells even with our proposed aggregation mechanism. Accordingly, the current protocols for cell membrane coating still have room for improvement. Considering the fact that only a median of 0.7% of the administered NPs are successfully delivered to solid tumors^51^, improved biomimetic designs could favor tumor uptake. The outcome of this study highlights some of the current limitations of cell membrane coating, especially the difficulty in separating cell membrane-coated NPs from the resulting mixture containing uncoated NPs, partially coated NPs and fully coated NPs. Hence, it would motivate the further studies on how to achieve a full cell membrane coating to improve targeting. Our quantification method to measure ratio of full coating allows to assess the success of these efforts and can serve as a reference standard for the different designs proposed in the literature. More importantly, if we are to further advance cell membrane coating technology, future translational research and fundamental investigations should move towards quantitative (e.g., examining the relationship between ratio of full coating and targeting efficiency), and away from qualitative results^52^.

In summary, we proposed a fluorescence quenching assay that is applicable for evaluating the cell membrane integrity of the biomimetic NPs. With this approach, we measured and compared the ratio of full coating of biomimetic NPs prepared with different parameters, and revealed that the current biomimetic design of cell membrane coating fails to obtain an adequate cell membrane integrity. Furthermore, we demonstrated that such partially coated NPs were able to enter the source cancer cells via a cooperation mechanism based on appropriate aggregation of NPs. We expect that broad and careful evaluation of the ratio of full cell membrane coating with respect to tumor targetability would provide a powerful, and generally applicable method to advance a new generation of cell membrane coating approaches to synthesize biomimetic NPs for cancer treatment.

## Methods

### Cell membrane derivation

The preparation of cell membrane materials with different cell types (CT26 cells, HeLa cells, RAW 264.7 macrophage cells) was performed according to a previously reported work with a slight modification^25^. Briefly, source cells were grown in 150 mm plates to full confluency and detached with Trypsin-EDTA solution and washed in Hank’s balanced salt solution (HBSS) three times by centrifuging at 1200 × *g*. The cells were suspended in a hypotonic lysing buffer consisting of 20 mM Tris-HCl pH = 7.5, 10 mM KCl, 2 mM MgCl_2_, and 1 EDTA-free mini protease inhibitor tablet per 10 mL of solution and disrupted using a dounce homogenizer with a tight-fitting pestle. The entire solution was subjected to 50 passes before spinning down at 3200 × *g* for 5 min. The supernatant was saved while the pellet was resuspended in hypotonic lysing buffer and subjected to another 50 passes and spun down again. The supernatants were pooled and centrifuged at 20,000 × *g* for 20 min, after which the pellet was discarded, and the supernatant was centrifuged again at 100,000 × *g* for 1 h. The pellet containing the plasma membrane material was then washed once in 10 mM HEPES pH = 7.5. The final pellet was collected and used as purified cancer cell membrane in the subsequent experiments. Red blood cell and platelet membrane materials were collected from male mice (6–8 weeks, Lab Animal Centre of University of Eastern Finland) according to the published hypotonic methods and repeated freeze-thaw process, respectively^53^. The bicinchoninic acid assay (BCA) protein assay was used to analyze the total protein content in the purified cell membrane. In this work, ~50 million cancer cells were able to obtain 2.5 mg membrane material (total protein weight).

### Preparation of core materials

In a typical synthesis of mesoporous SiO_2_ NPs, 6.24 g hexadecyltrimethylammonium bromide (CTAB) was dissolved in 53.4 mL deionized water, followed by adding 0.3 g sodium acetate trihydrate. After stirring for 2 h at 60 °C, 4.35 mL tetraethyl orthosilicate (TEOS) was added dropwise into solution under vigorous stirring, and then heated at 60 °C for 12 h. Afterwards, the resultant colloid was collected, washed with ethanol, and then transferred into a mixture of ethanol (100 mL) and concentrated hydrochloric acid (2.0 mL) with continual stirring at 90 °C for 24 h to remove surfactant. The extracted NPs were washed with ethanol three times and resuspended in absolute ethanol.

Spherical nonporous SiO_2_ NPs were synthesized by a previously reported Stöber method^54^. The size of nonporous SiO_2_ NPs was controlled by changing the amounts of ammonium hydroxide (Supplementary Table 1). The resulting nonporous SiO_2_ NPs were collected by centrifugation and washed with ethanol three times to remove unreacted precursors.

### Synthesis and characterization of cell membrane-coated NPs

In the preparation of cell membrane-coated NPs, membrane material derived as described above was first physically extruded through a 400 nm polycarbonate membrane for 13 passes to create cell membrane-derived vesicles. Then, the resulting vesicles were coated onto NPs by sonication or co-extruding vesicles and cores through a 200 nm polycarbonate membrane. In order to measure the ratio of full coating, the bare core NPs needed to be replaced by the NBD-labeled NPs. The as-synthesized cell membrane-coated NPs were further purified by centrifugation to remove any excess free cell membrane.

In the transmission electron microscopy (TEM) characterization, one drop of prepared particles was left on the grid for 1 min before being washed off with 3 drops of water. The grids were then negatively stained with 1 drop of 1% uranyl acetate. Excess solution was wiped away with absorbent paper and the samples were imaged using a JEM-2100F (JEM Ltd., Japan) microscope. The hydrodynamic diameter and zeta potential were measured by a dynamic light scatter (DLS; Malvern Zetasizer Nano ZS). Sodium dodecylsulfate polyacrylamide gel electrophoresis (SDS-PAGE) analysis was conducted to characterize the protein composition. Stability experiments were performed by measuring NPs in 1X PBS for 6 days using DLS.

### Calculation of ratio of full cell membrane coating

The fluorescence intensity was monitored over time on a Synergy H1 microplate reader (Biotek, Winooski, USA; *λ*_exc_ = 465 nm). After a baseline was established, 20 µL 1 mol/L sodium dithionite solution was added to the prepared samples (100 µL). Data were normalized with the average fluorescence obtained for cell membrane-coated core materials. The ratio of full coating was calculated from the reserved fluorescence, using the following equations:

$${{{{{\rm{Ratio}}}}}}\; {{{{{\rm{of}}}}}}\; {{{{{\rm{full}}}}}}\; {{{{{\rm{coating}}}}}}\,( \% )=\left[\left({F}_{D}-{F}_{0}\right)/\left({F}_{T}-{F}_{0}\right)\right]\times 100$$

where *F*_T_ is the total fluorescence of the sample before addition of dithionite, *F*_D_ is the fluorescence of the sample following quenching with dithionite and *F*_0_ is the fluorescence of background.

### Cell culture

CT26 cells were cultured with RPMI-1640 supplemented with 10% fetal bovine serum (FBS) and 1% antibiotic antimycotic solution (100×). MCF-7 cells, HeLa cells and RAW 264.7 macrophage cells were cultured in DMEM medium containing 10 % FBS and 1% antibiotic antimycotic solution (100×). All the cells were cultured at 37 °C in a humidified atmosphere with 5% CO_2_.

### Cell viability

The cytotoxicity of the SiO_2_ NPs, LB-SiO_2_ NPs and CM-SiO_2_ NPs was examined by CellTiter-Glo Luminescent Cell Viability Assay (Promega Co.). CT26 cells were each seeded in a 96-well plate at a density of 1×10^4^ cells/well and cultured for 24 h in 100 μL RPMI-1640 medium containing 10% FBS. The medium was discarded and replaced with 100 μL fresh medium containing various concentrations of SiO_2_ NPs, LB-SiO_2_ NPs and CM-SiO_2_ NPs and no additive (negative control). After 24 h incubation at 37 °C, 100 μL CellTiter-Glo reagent was added. The contents were mixed, and the plate was allowed to incubate at room temperature for 10 min to stabilize the luminescence signals. The cell viability was detected from the luminescent intensity, which represents the amount of ATP produced by the viable cells. The assay was carried out using Synergy H1 microplate reader (Biotek, Winooski, USA). All samples were performed in eight replicates.

### Homotypic targeting studies

Confocal laser scanning microscopy (CLSM), flow cytometric assay (FCA) and TEM were used to investigate the homotypic targeting ability of CM-SiO_2_ NPs. For CLSM observation, different cell lines were seeded in μ-Slide 8 well Ibidi imaging plates (Ibidi GmbH, Germany) at a density of 2.5 × 10^4^ cells per well and cultured for 24 h in 200 µL of DMEM or RPMI-1640 medium containing 10% FBS and 1% antibiotic antimycotic solution (100×). Afterwards, the medium was later replaced with a fresh medium containing SiO_2_ NPs, LB-SiO_2_ NPs and CM-SiO_2_ NPs (50 μg/mL), respectively. After 4 h coincubation, the medium was removed and the cells were washed three times with HBSS. After further incubation in 200 μL of DAPI solution (1 μg/mL in HBSS) for 10 min at room temperature, the cells were photographed by CLSM (Zeiss LSM 700, Carl Zeiss, Jena, Germany).

In the flow cytometric analysis, Cy5-labeled SiO_2_ NPs were used to track the uptake of the CM-SiO_2_ NPs. Initially, different cell lines were seeded in 6-well plates at a density of 5 × 10^5^ cells per single well and cultured for 24 h in 2 mL of DMEM or RPMI-1640 medium containing 10% FBS and 1% antibiotic antimycotic solution (100×). After SiO_2_ NPs, LB-SiO_2_ NPs and CM-SiO_2_ NPs (50 μg/mL) were coincubated with the cells for 4 h, then the cells were washed three times with HBSS, detached by trypsin-EDTA and finally collected by centrifugation at 1200 × *g* for 5 min. The bottom cells were washed three times with HBSS and then the suspended cells were analyzed by flow cytometry. In this study, the gating strategy used to quantify nanoparticle binding and uptake was presented in Supplementary Fig. 28.

For TEM observation, the different cell lines were cultured and collected as described in flow cytometric analysis. After the collected cells washed three times with HBSS, 1 mL/well of 2.5% glutaraldehyde was added to the cells and incubated at 37 °C for 20 min for cell fixation, followed by washing with HBSS for three times. Fixed cells were embedded in 2% agarose, rinsed with distilled water and dehydrated with ethanol (80%–100%). Finally, the cells were processed for embedding in Epon, by polymerizing 8 h at 37 °C and 56 h at 60 °C, and then cut into 70–100 nm thick slices. After polymerization, the cells were contrasted with uranyl acetate and lead citrate. The samples were imaged using a JEM-2100F (JEM Ltd., Japan) microscope.

### Endocytosis pathway inhibition

To study the endocytosis mechanism for CM-SiO_2_ NPs, the cells were pre-incubated with the following inhibitors for 2 h at 37 °C: 10 μg/mL chlorpromazine (clathrin-mediated endocytosis inhibitor), 200 μM genistein (caveolin-mediated endocytosis inhibitor), 2.5 μg/mL cytochalasin D (macropinocytosis inhibitor). Alternatively, the cells were placed at 4 °C to investigate the effect of temperature on the internalization of CM-SiO_2_ NPs. After that, the medium was removed, and the freshly prepared test complexes (Cy5-labeled CM-SiO_2_ NPs) in media containing inhibitors at the same concentrations were added and further incubated for 4 h. Then, the cells were washed three times with HBSS, collected according to the methods described above and analyzed via flow cytometry to assess the uptake of the CM-SiO_2_ NPs. In this experiment, the group without any treatment was used as a background in the flow cytometry analysis, while the group in the presence of CM-SiO_2_ NPs but without inhibitor treatment was used as a control. The endocytosis inhibition was quantified by normalizing the geometric mean fluorescence of wells treated with inhibitor to that of control wells.

### Course-grained model

Supplementary Fig. 17 illustrates the setup and the different components of the DPD model used in our simulations. The CM-SiO_2_ NP consisted of coated part (orange) and uncoated part (blue). For simplification, the thickness and the flexibility of the coated cell membrane of the CM-SiO_2_ NPs were not considered. The whole NP of a diameter of 15 r_c_ was fixed as a rigid body during the simulations. The NP was placed 1 r_c_ above the membrane initially. The lipid model was presented by the H_3_T_5_ model proposed by Groot and Rabone^55^. The bonded and unbonded parameterization of the lipid model were adopted from references^56,57^. The DPD parameters between the SiO_2_ NP and the lipids were refereed from previous report^58^, which results in a weak adhesion of the SiO_2_ NP on the lipid membrane (Fig. 4a). The parametrization of the whole system (*a*_*ij*_) was summarized in Supplementary Table 2. Half of the lipid heads of the membrane were considered as the receptors, where a high ratio of receptors has been proved an efficient way to simulate the receptor-mediated endocytosis^59^. The coated parts of the NP were considered as the ligands, which can specifically interact with the cell receptors. During the simulations, the area per lipid of the membrane was adjusted to about 1.1 r_c_^2^ by compressing or stretching the simulation box, which can keep the membrane at a zero-surface tension^57,60^.

### Statistics and reproducibility

All the experiments including those in Supplementary Information were performed at least three times independently with similar results. Results are expressed as mean ± SD. The data were analyzed for statistical significance by one-way analysis of variance (ANOVA) followed by post hoc Tukey test. Statistical analyses were performed using Origin 2019 software (OriginLabs). *p* < 0.05 was considered statistically significant.

### Reporting summary

Further information on research design is available in the Nature Research Reporting Summary linked to this article.

## Supplementary information

Supplementary Information

Description of Additional Supplementary Files

Supplementary Movie 1

Supplementary Movie 2

Supplementary Movie 3

Reporting Summary

## Data Availability

The authors declare that all data supporting the findings of this study are available within the paper and its supplementary information files or available from the corresponding author upon reasonable request.
