# Supplementary material for: Cell membrane coating integrity affects the internalization mechanism of biomimetic nanoparticles
Source: Nat Commun. 2021 Sep 30;12:5726. doi: 10.1038/s41467-021-26052-x (PMC8484581; doi:10.1038/s41467-021-26052-x)
Supplement: Supplementary file 1 — Supplementary Information [file 41467_2021_26052_MOESM1_ESM.pdf]

Supplementary Information for:

Cell membrane coating integrity affects the internalization mechanism of biomimetic nanoparticles

Lizhi Liu[1,7], Xuan Bai[2,7], Maria-Viola Martikainen[3], Anna Kårlund[4], Marjut Roponen[3], Wujun Xu[1]*, Guoqing Hu[2], Ennio Tasciotti[5,6] and Vesa-Pekka Lehto[1]*

[1]Department of Applied Physics, University of Eastern Finland, 70210 Kuopio, Finland
[2]Department of Engineering Mechanics, State Key Laboratory of Fluid Power and Mechatronic Systems, Zhejiang University, Hangzhou 310027, China
[3]Department of Environmental and Biological Sciences, University of Eastern Finland, 70210 Kuopio, Finland
[4]Institute of Public Health and Clinical Nutrition, University of Eastern Finland, 70211 Kuopio, Finland
[5]IRCCS San Raffaele Pisana Hospital and San Raffaele University, Rome, Italy
[6]Sclavo Pharma, Siena, Italy
[7]These authors contributed equally: Lizhi Liu, Xuan Bai
*Corresponding author: wujun.xu@uef.fi, vesa-pekka.lehto@uef.fi

Supplementary Methods

Materials

Tetraethyl orthosilicate (TEOS), hexadecyltrimethylammonium bromide (CTAB), Au NPs (size: 80 nm), fetal bovine serum (FBS), antibiotic antimycotic solution (100×), trypsin-EDTA, 4',6-diamidino-2-phenylindole (DAPI), 1,1′-dioctadecyl-3,3,3′,3′-tetramethylindocarbocyanine perchlorate (DiI) and bovine serum albumin (BSA) were purchased from Sigma-Aldrich Corporation (St Louis, MO, USA). Cyanine 5 (Cy5) was purchased from Lumiprobe Corporation (Hallandale Beach, FL, USA). 1,2-dioleoyl-sn-glycero-3-phosphocholine (DOPC) and 1,2-distearoyl-sn-glycero-3-phosphoethanolamine-N-(7-nitro-2-1,3-benzoxadiazol-4-yl) (NBD-DSPE) were purchased from Avanti Polar Lipids Inc. (Alabaster, AL, USA). Bicinchoninic acid (BCA) assay kit and 6-(7-Nitrobenzofurazan-4-ylamino)dodecanoic acid NHS ester (NBD-X) were purchased from Thermo Fisher Scientific (Waltham, MA). Dulbecco's modified Eagle's medium (DMEM) and RPMI 1640 medium were purchased from Biowest Corporation, France. Phosphate buffer saline (PBS) and Hank's balanced salt solution (HBSS) were obtained from HyClone. CellTiter-Glo assay was purchased from Promega Corporation, USA. ZIF-8 NPs[1], Fe_3O_4 NPs[2], poly(lactic-co-glycolic acid) (PLGA) NPs[3] and porous silicon (PSi) NPs[4] were synthesized according to the literatures. All chemical reagents were used directly without further purification unless specifically mentioned.

Fluorescence labeling

NBD labeled core materials (mesoporous SiO_2 NPs, nonporous SiO_2 NPs, Fe_3O_4 NPs, and PSi NPs) were obtained through sequential chemical surface modification. Briefly, 50 mg core NPs were reacted with 20 uL APTES in 10 mL ethanol at room temperature for 2 h. The as-synthesized amine-modified NPs were washed with ethanol twice and then reacted with NBD in ethanol at room temperature overnight. To make a negative surface charge of NPs on the cell membrane coating, the excess amine groups were reacted with succinic anhydride in ethanol. After rinsing with ethanol twice, the NBD labeled were successfully harvested. In the preparation of fluorescent PLGA NPs, NBD was covalently attached to the polymers by a carbodiimide method[5]. In order to obtain NBD labeled ZIF-8 NPs, NBD was encapsulated in the ZIF-8 NPs during the biomimetic growth process. The NBD labeled Au NPs were prepared by mixing the Au NPs with dye under stirring for 2 h and then washed three times with deionized water.

Preparation of GUVs, LB-SiO_2 NPs, E-SiO_2 NPs, Ex-SiO_2 NPs and BSA adsorbed NPs

Giant unilamellar vesicles (GUVs) comprising DOPC/NBD-DSPE (molar ratio: 150/1) were prepared by a natural swelling method as follows[6]. First, the above lipid mixture in chloroform was dried under nitrogen gas to obtain a thin, homogeneous lipid film. Subsequently, the residual chloroform in the film was completely removed by placing the bottle in a vacuum desiccator for 12 h. Then, 100 μL of buffer containing 20 mM HEPES (pH 7.0), 150 mM NaCl and 100 mM sucrose were added to the resulting lipid film and pre-hydrated at 45 °C for 10 min. After that, a 900 μL aliquot of the same buffer was added, and the glass bottle was resealed and incubated in an incubator at 37 °C for 2 h to produce the GUV suspension. The morphology of GUVs was observed under a confocal laser scanning microscopy (CLSM; Zeiss LSM 700, Carl Zeiss, Jena, Germany). The control membrane, DOPC lipid bilayer coated SiO_2 (LB-SiO_2) NPs, were prepared according to a procedure already described[7].

To obtain endocytosed SiO_2 (E-SiO_2) NPs, CT26 cells were treated with 200 μg/mL positively charged SiO_2 NPs for 12 h in 15 cm dishes. Then, the cells were detached with Trypsin-EDTA solution and washed by HBSS for three times. The as-prepared E-SiO_2 NPs were collected by centrifuging at 1200 × g. With respect to exocytosed SiO_2 (Ex-SiO_2) NPs, after the CT26 cells had been incubated with 200 μg/mL positively charged SiO_2 NPs for 24 h in 15 cm dishes, the cell media were discarded and replaced with fresh media. After a further 12 h incubation, the cells were

discarded at 1200 g for 5 min and then the supernatants were further centrifuged at 10,000 g for 15 min to obtain Ex-SiO_2 NPs. To prepare BSA adsorbed NPs, both the positively charged NPs and negatively charged NPs (1 mg/mL) were incubated with BSA solution (1 mg/mL) at 37 °C for 4 h, and then the superfluous BSA was discarded by centrifugation.

DPD formulation

The dissipative particle dynamics (DPD) technique was used to study the cellular pathways of the CM-SiO_2 NPs, which has been widely applied in the studies of the NP-membrane interactions[8-10]. In the DPD simulations, a cluster of atoms can be represented by a single bead and each bead is governed by the Newton's equation of motion,

$$\frac{dv_i}{dt} = \frac{F_i}{m} \tag{1}$$

where the mass *m* of per bead is set to 1. The total force between beads *i* and *j* consists of three types of forces: conservative force F_{ij}^{C}, dissipative force F_{ij}^{D}, and random force F_{ij}^{R}. The total force on the bead *i* can be expressed as,

$$F_i = \sum_{i \neq j} F_{ij}^C + F_{ij}^D + F_{ij}^R \tag{2}$$

where the sum runs over all beads *j* within a certain cutoff radius r_c. The conservative force is a soft-repulsive force taken as,

$$F_{ij}^C = a_{ij}(1 - r_{ij})\mathbf{r}_{ij} \tag{3}$$

where a_{ij} is the maximum repulsion between the bead *i* and *j*, $r_{ij} = \left|\mathbf{r}_i - \mathbf{r}_j\right| / r_c$, and $\mathbf{r}_{ij} = \mathbf{r}_{ij} / \left|\mathbf{r}_{ij}\right|$. The dissipative force is given by,

$$F_{ij}^D = -\gamma\omega_D(r_{ij})(\mathbf{r}_{ij} \cdot \mathbf{v}_{ij})\mathbf{r}_{ij} \tag{4}$$

Where γ is the strength of friction, $\omega_D(r_{ij}) = (1 - r_{ij})^2$ and $\mathbf{v}_{ij} = \mathbf{v}_i - \mathbf{v}_j$. The random force is,

$$F_{ij}^R = \sigma\omega_R(r_{ij})\xi_{ij}\mathbf{r}_{ij} \tag{5}$$

where ξ_{ij} is a zero-mean Gaussian random variable of unit variance, $\omega_R(r_{ij}) = 1 - r_{ij}$, and σ is a noise amplitude that equals to $\sqrt{2k_BT\gamma}$ [11]. We take r_c as the characteristic length scale and k_BT as the characteristic energy scale. The characteristic time scale is defined as $\tau = \sqrt{mr_c^2 / k_BT}$. Equation (1) is integrated in time with a velocity-Verlet algorithm at $\sigma = 3$ and $\Delta t = 0.01\ \tau$ [12]. Two simulation systems were used here: the smaller system ($40\times100\times100\ r_c^3$) was used for studying the 1, 2, 4 NPs aggregates and performed at $8\times10^4\ \tau$; the larger system ($40\times150\times150\ r_c^3$) was used for studying the 9,16 NPs aggregates and performed for $1.6\times10^5\ \tau$. The periodic boundaries are applied in all the x, y, z directions of the simulation box. The bead density of each simulation box is a constant of 3. All simulations were performed in the NVT ensembles using the LAMMPS package[13].

Supplementary Figures

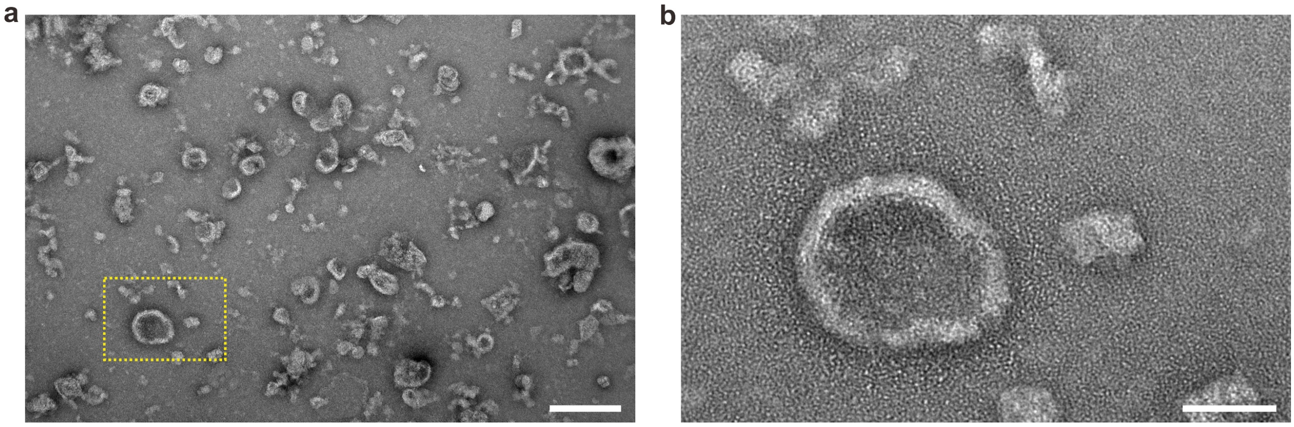

Supplementary Fig. 1 TEM images of cell membrane vesicles at low-magnification (**a**) and high-magnification (**b**). Scale bars, 200 nm in (a) and 50 nm in (b).

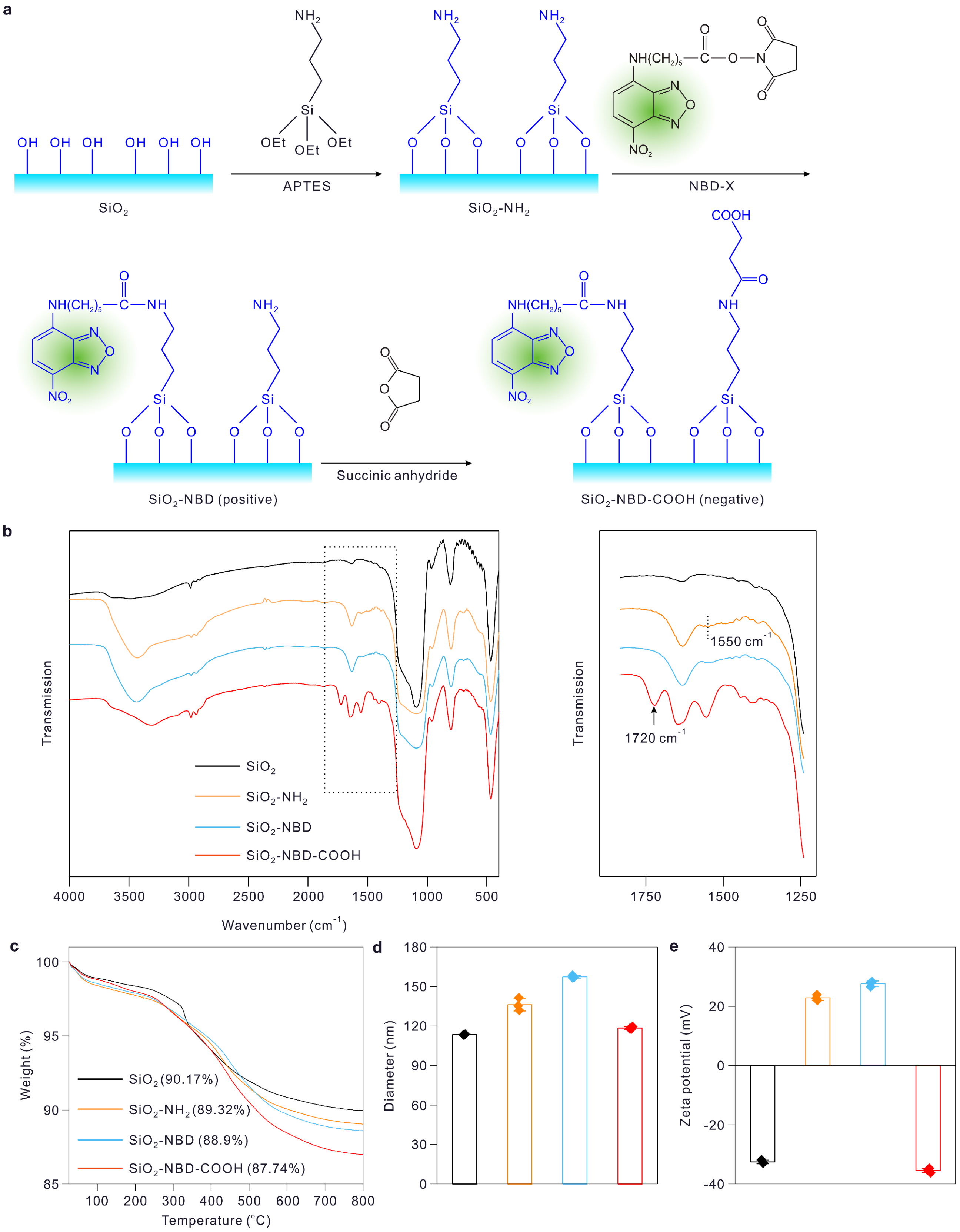

Supplementary Fig. 2 a, NBD labeled NPs were achieved by surface chemical modification of the NPs with 3-aminopropyltriethocysilane (APTES), NBD dodecanoic acid N-succinimidyl ester (NBD-X), and succinic anhydride, respectively. **b-e,** Fourier transform infrared spectroscopy (FTIR, Thermo Nicolet iS 50) spectra (**b**), thermogravimetric analysis (TGA; **c**), mean diameter (**d**) and zeta potential (**e**) of SiO_2, SiO_2-NH_2, SiO_2-NBD, and SiO_2-NBD-COOH NPs. The characteristic peak for amine

modification was observed at 1550 cm^{-1} owing to $-NH_2$ bending vibration. The characteristic absorption at 1720 cm^{-1} indicated that there are carboxylic groups on the final product (SiO_2-NBD-COOH). Due to only 0.42% of NBD was grafted onto SiO_2 NPs calculated from thermogravimetric analysis (TGA) results (c), the absorptions of NBD were difficult to be detected from FTIR. Alternatively, the fluorescence images of NBD labelled SiO_2 NPs (Fig. 1k) supported that the NBD was successfully modified onto the SiO_2 NPs. These modifications were further confirmed by the measurement of size and zeta potential, in which the zeta potential changed from -32.5 mV to 22.9 mV after amine modification. Data represents mean ± SD (n = 3).

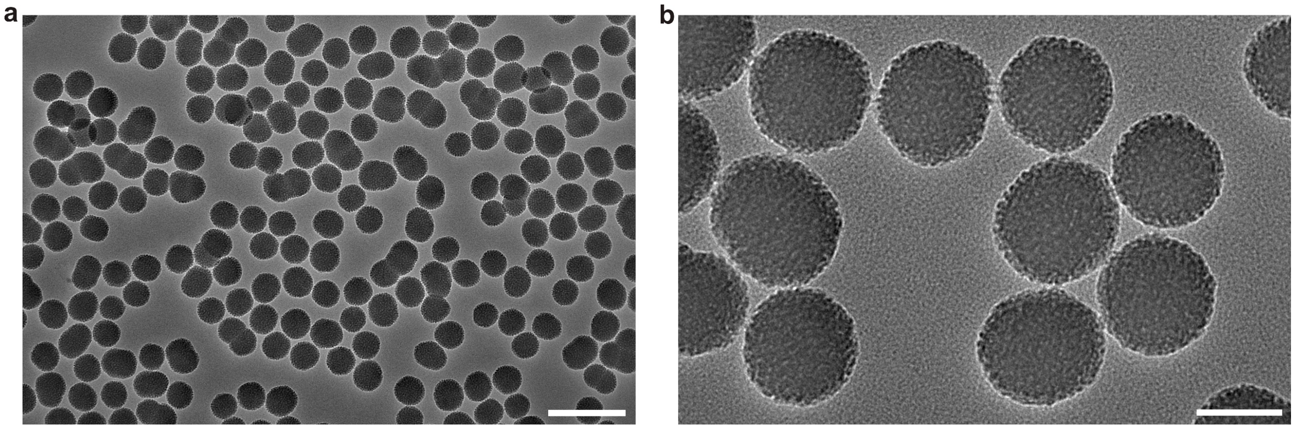

Supplementary Fig. 3 TEM images of mesoporous SiO_2 NPs at low-magnification (**a**) and high-magnification (**b**). Scale bars, 200 nm in (a) and 50 nm in (b).

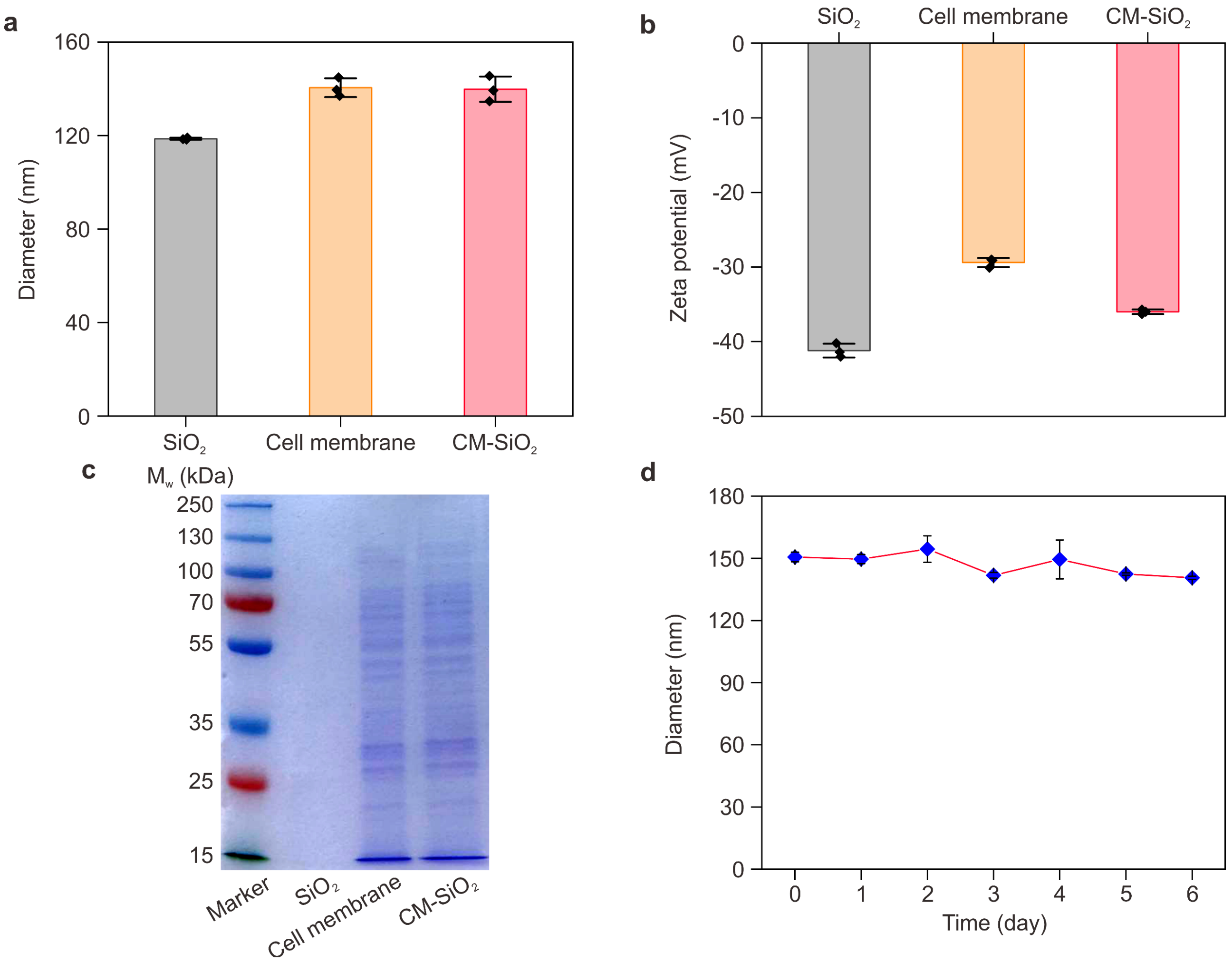

Supplementary Fig. 4 Physicochemical characterization of SiO_2 NPs, cell membrane vesicles and CM-SiO_2 NPs. **a,b,** Mean diameter (**a**) and zeta potential (**b**) of SiO_2 NPs, cell membrane vesicles and CM-SiO_2 NPs. Data represents mean ± SD ($n = 3$). **c**, SDS-PAGE protein analysis of SiO_2 NPs, cell membrane vesicles and CM-SiO_2 NPs. **d**, Stability of CM-SiO_2 NPs in 1X PBS, determined by monitoring the particle size (diameter, nm), over a span of 6 day. Data represents mean ± SD ($n = 3$).

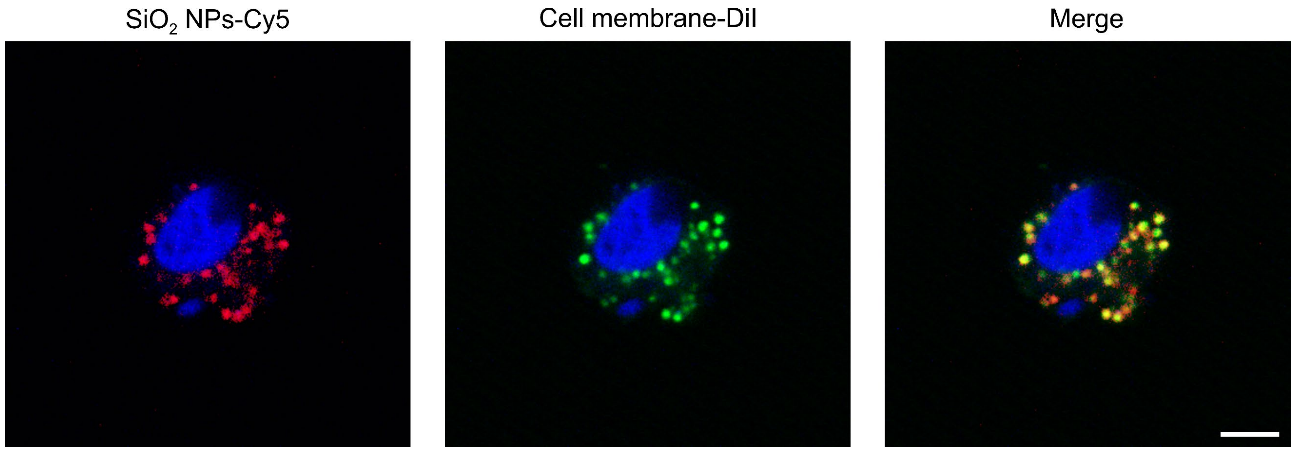

Supplementary Fig. 5 Confocal laser scanning microscopy (CLSM) images showed the colocalization of the SiO_2 NPs (labeled with Cy5; red) and cell membranes (labeled with DiI; green) after being internalized by CT26 cells. The CM-SiO_2 NPs were incubated with CT26 cells for 4 h. The cell nuclei were stained with DAPI (blue). Scale bar, 5 μm.

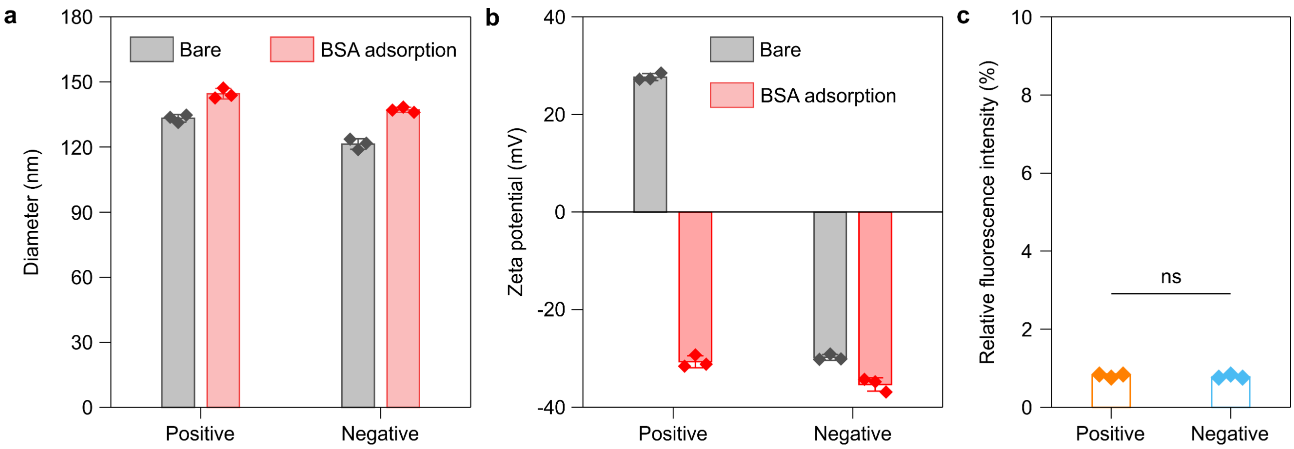

Supplementary Fig. 6 a,b, Mean diameter (**a**) and zeta potential (**b**) of positive charge and negative charge functionalized SiO_2 NPs before and after adsorption of BSA. **c,** Relative fluorescence intensity of positively charged and negatively charged NPs after addition of quencher. Data represents mean ± SD ($n = 3$). One-way ANOVA followed by post hoc Tukey test was used to determine the significance of data. ns: not significant.

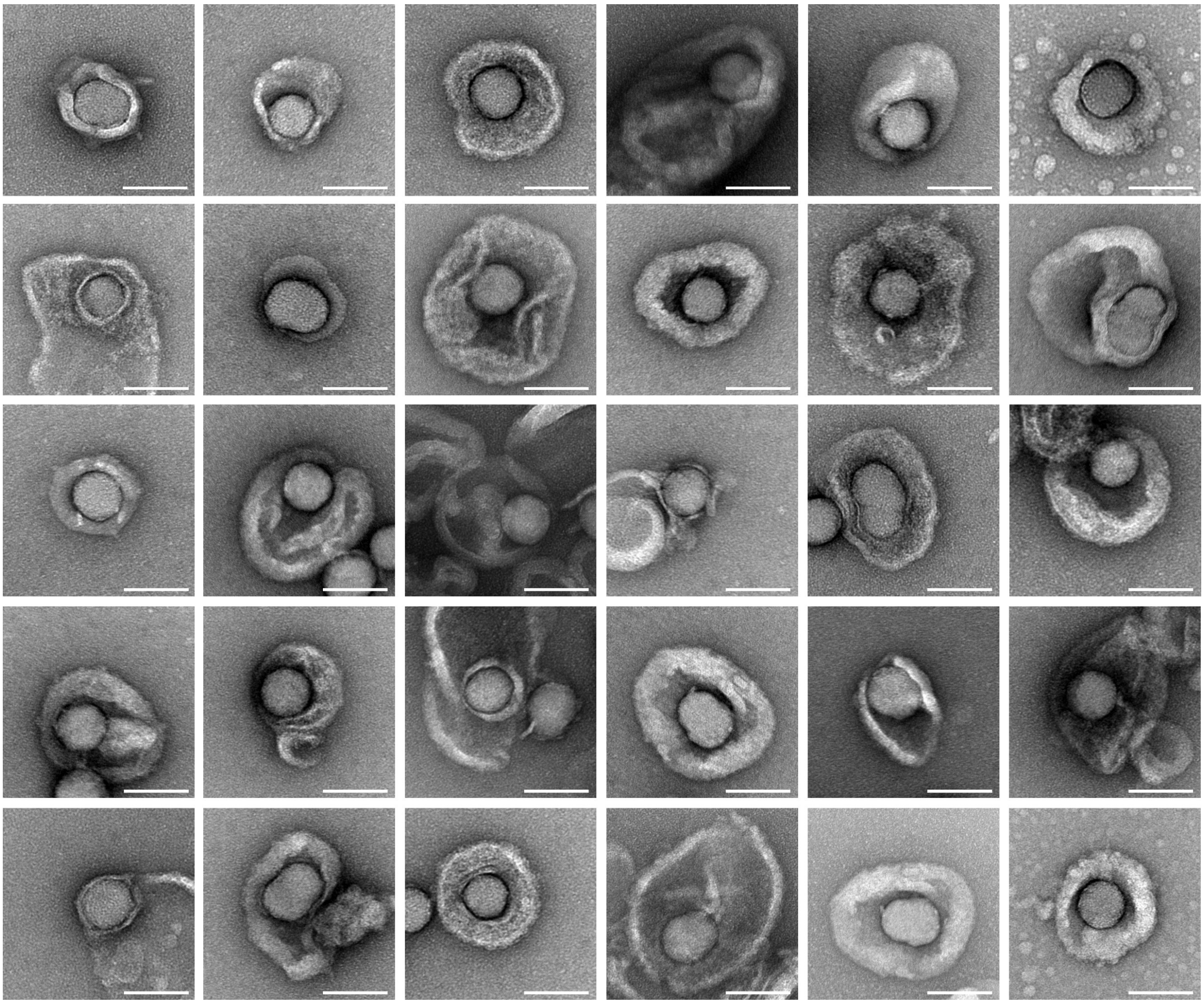

Supplementary Fig. 7 TEM images of cell membrane fully coated mesoporous SiO_2 NPs. Scale bars, 100 nm. According to these TEM images, the full cell membrane coating can be classified to two different types: tight and loose, depending on the size of encountered cell membrane vesicles during the coating process. Specifically, when the size of cell membrane vesicles is comparable to that of SiO_2 NPs, the SiO_2 NPs can be wrapped tightly by the cell membrane. By contrast, when the size of cell membrane vesicles is significantly bigger than that of SiO_2 NPs, the SiO_2 NPs are coated loosely.

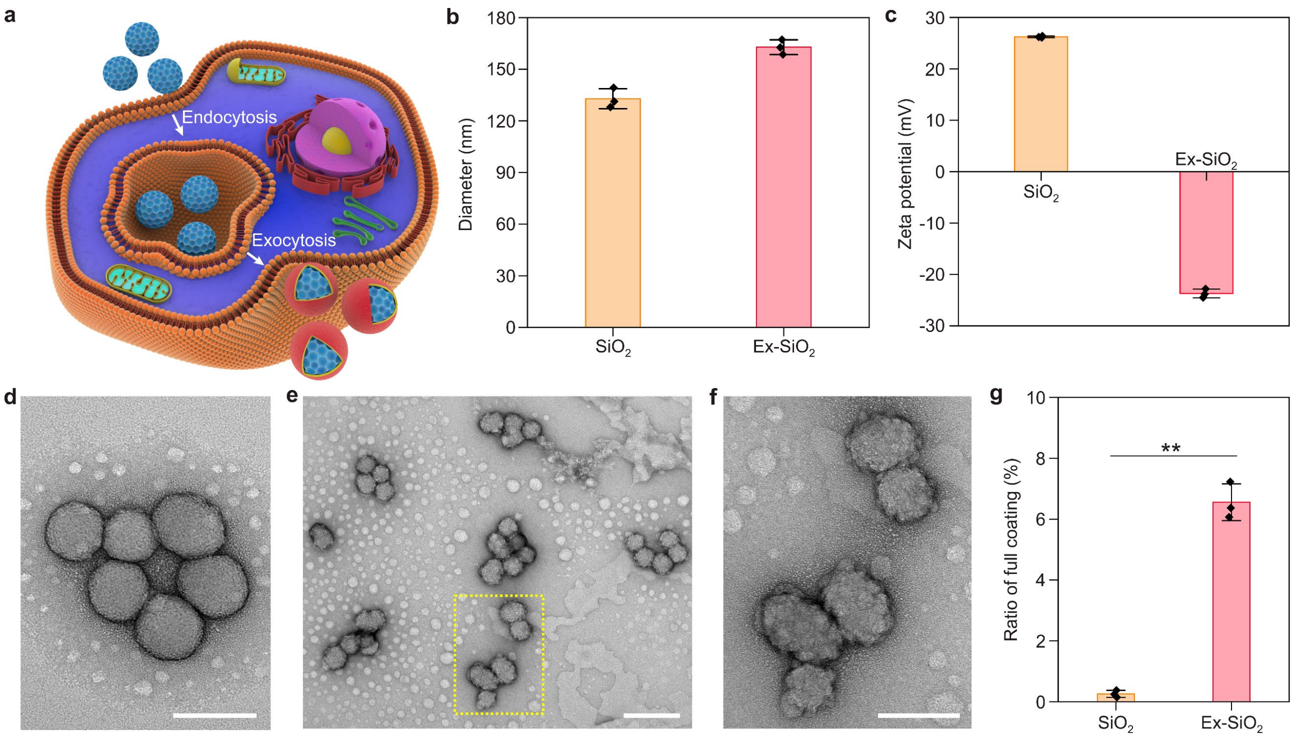

Supplementary Fig. 8 **a**, Schematic illustration of the preparation of Ex-SiO_2 NPs. Positively charged mesoporous SiO_2 NPs are endocytosed into CT26 cancer cells and then exocytosed into the extracellular space after incubation. **b,c,** Mean diameter (**b**) and zeta potential (**c**) of positively charged mesoporous SiO_2 NPs and Ex-SiO_2 NPs. **d-f,** TEM images of bare SiO_2 NPs (**d**) and Ex-SiO_2 NPs at low-magnification (**e**) and high-magnification (**f**). Scale bars, 100 nm in d and f and 200 nm in e. **g**, Quantification of the ratio of full cell membrane coating for Ex-SiO_2 NPs. Data represents mean ± SD ($n = 3$). One-way ANOVA followed by post hoc Tukey test was used to determine the significance of data. $p = 5.9E-5$. $^{**}p < 0.01$.

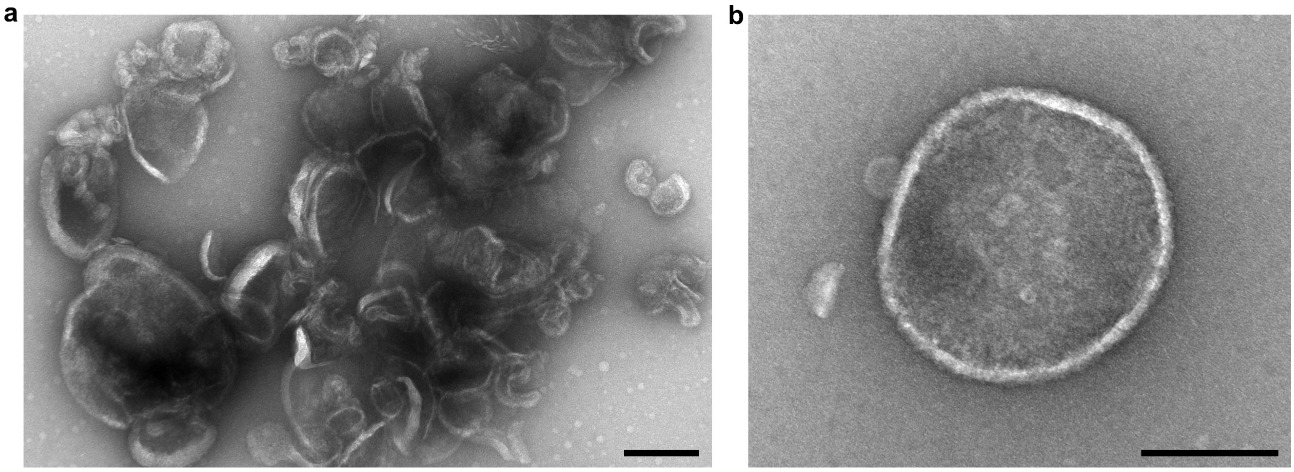

Supplementary Fig. 9 TEM images of RBC membrane vesicles at low-magnification (**a**) and high-magnification (**b**). Scale bars, 200 nm.

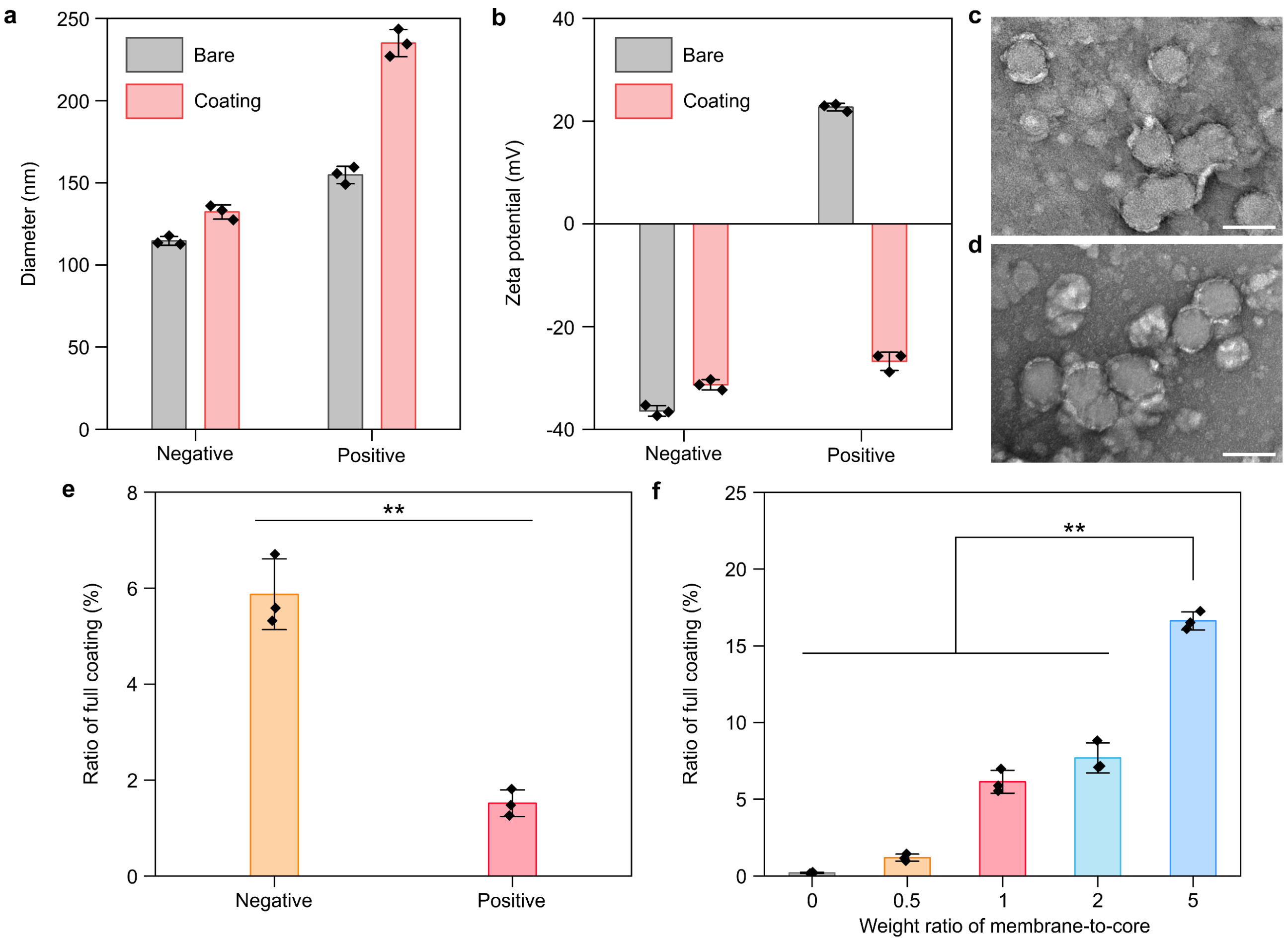

Supplementary Fig. 10 a,b, Mean diameter (**a**) and zeta potential (**b**) of negative charge and positive charge functionalized mesoporous SiO_2 NPs before and after coating with cell membranes. **c,d,** TEM images of negative charge (**c**) and positive charge (**d**) functionalized mesoporous SiO_2 NPs after coating with cell membranes. Scale bars, 100 nm. **e**, Quantification of the ratio of full cell membrane coating for negative charge and positive charge functionalized mesoporous SiO_2 NPs. **f**, Quantification of the ratio of full cell membrane coating with different weight ratios of cell membranes to core materials (SiO_2 NPs). Data represents mean ± SD ($n = 3$). One-way ANOVA followed by post hoc Tukey test was used to determine the significance of data in **e** and **f**. $p = 6.7E\text{-}4$ (**e**). $^{**}p < 0.01$.

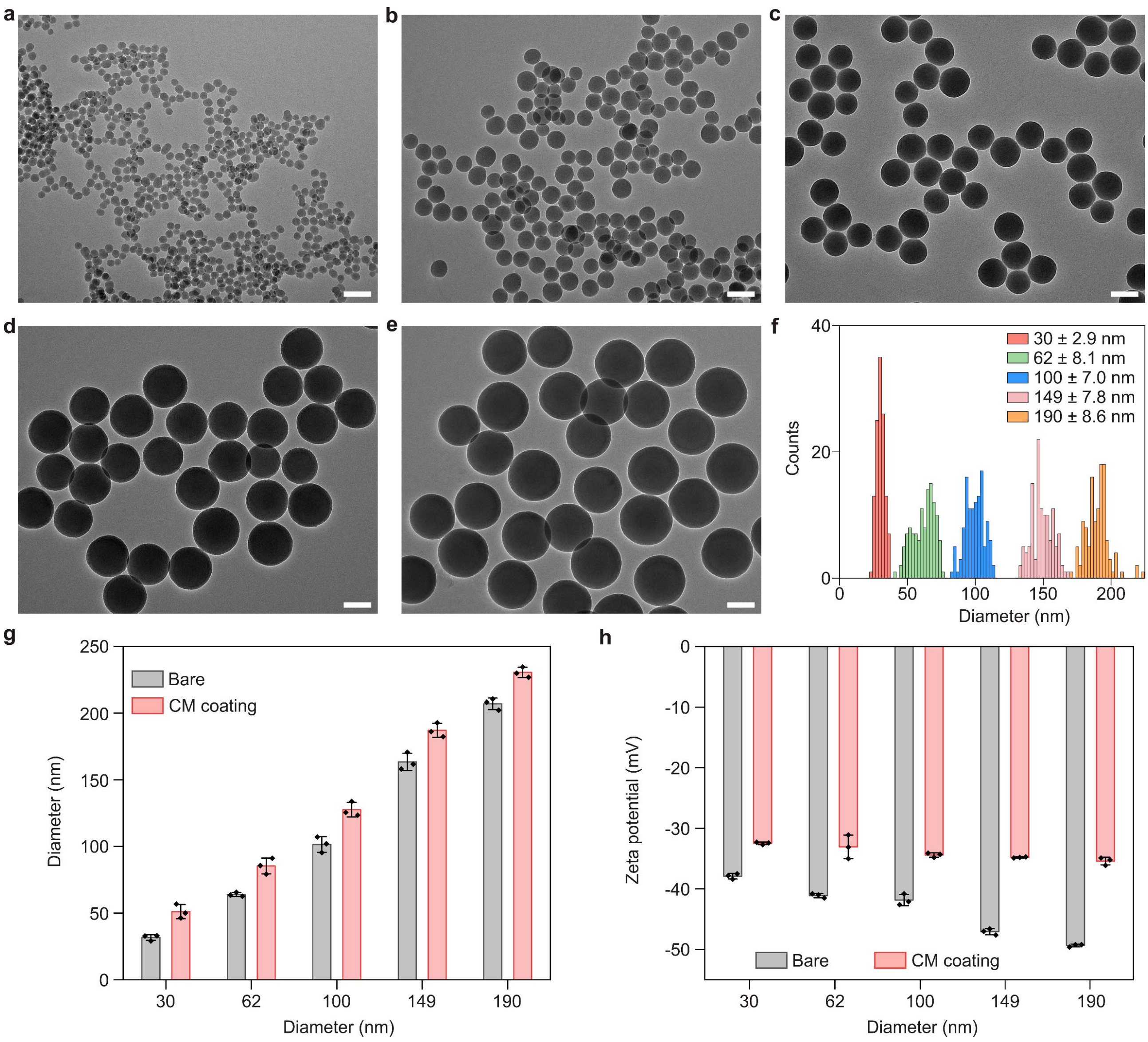

Supplementary Fig. 11 a−e, TEM images of nonporous Stöber SiO_2 NPs of varied sizes: 30 nm (**a**), 62 nm (**b**), 100 nm (**c**), 149 nm (**d**), and 190 nm (**e**). Scale bars, 100 nm. **f**, Particle size distributions of five sizes of nonporous Stöber SiO_2 NPs (n = 120). The data were calculated from TEM images. **g**,**h**, Mean diameter (**g**) and zeta potential (**h**) of nonporous Stöber SiO_2 NPs before and after coating with cell membranes. Data represents mean ± SD (n = 3).

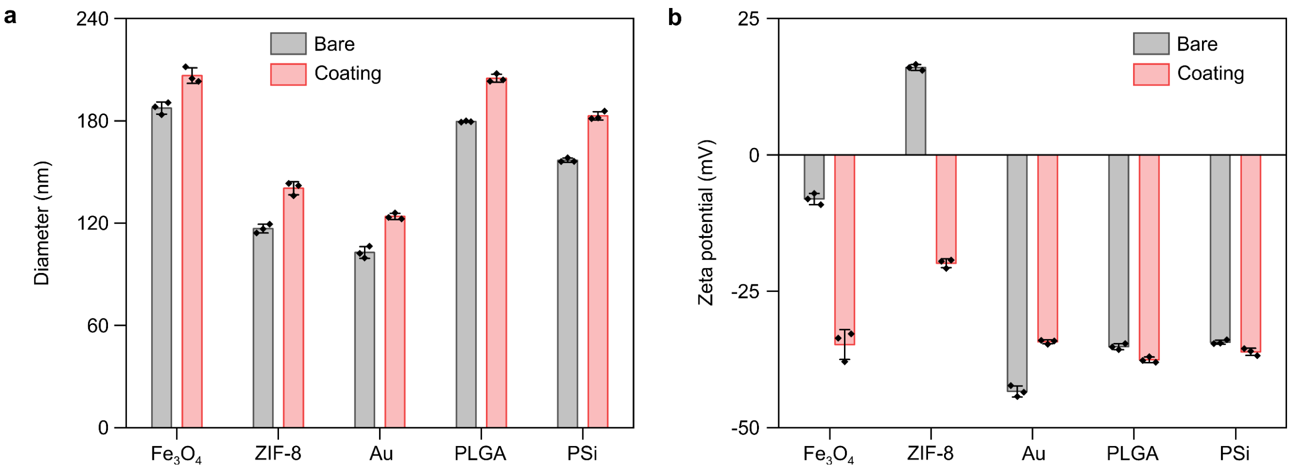

Supplementary Fig. 12 Mean diameter (**a**) and zeta potential (**b**) of Fe_3O_4 NPs, ZIF-8 NPs, Au NPs, PLGA NPs, and PSi NPs before and after coating with cell membranes. Data represents mean ± SD (n = 3).

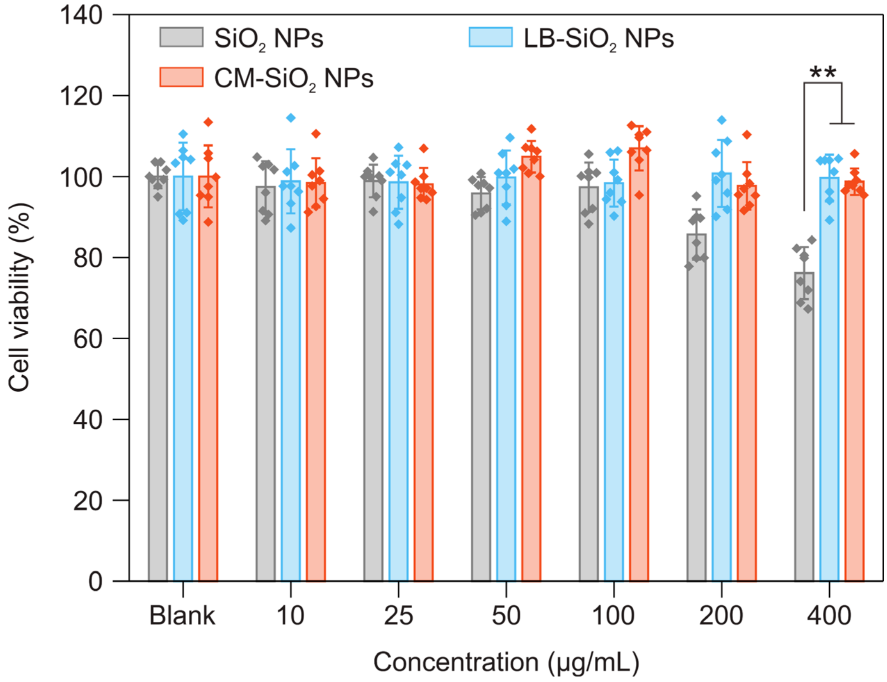

Supplementary Fig. 13 Cell viability of CT26 cells after incubation with SiO_2 NPs, LB-SiO_2 NPs and CM-SiO_2 NPs for 24 h at different concentrations. Data represents mean ± SD ($n = 8$). One-way ANOVA followed by post hoc Tukey test was used to determine the significance of data. $p = 1.7E\text{-}8$ (SiO_2 NPs vs. LB-SiO_2 NPs), $p = 6.3E\text{-}8$ (SiO_2 NPs vs. CM-SiO_2 NPs). $^{**}p < 0.01$.

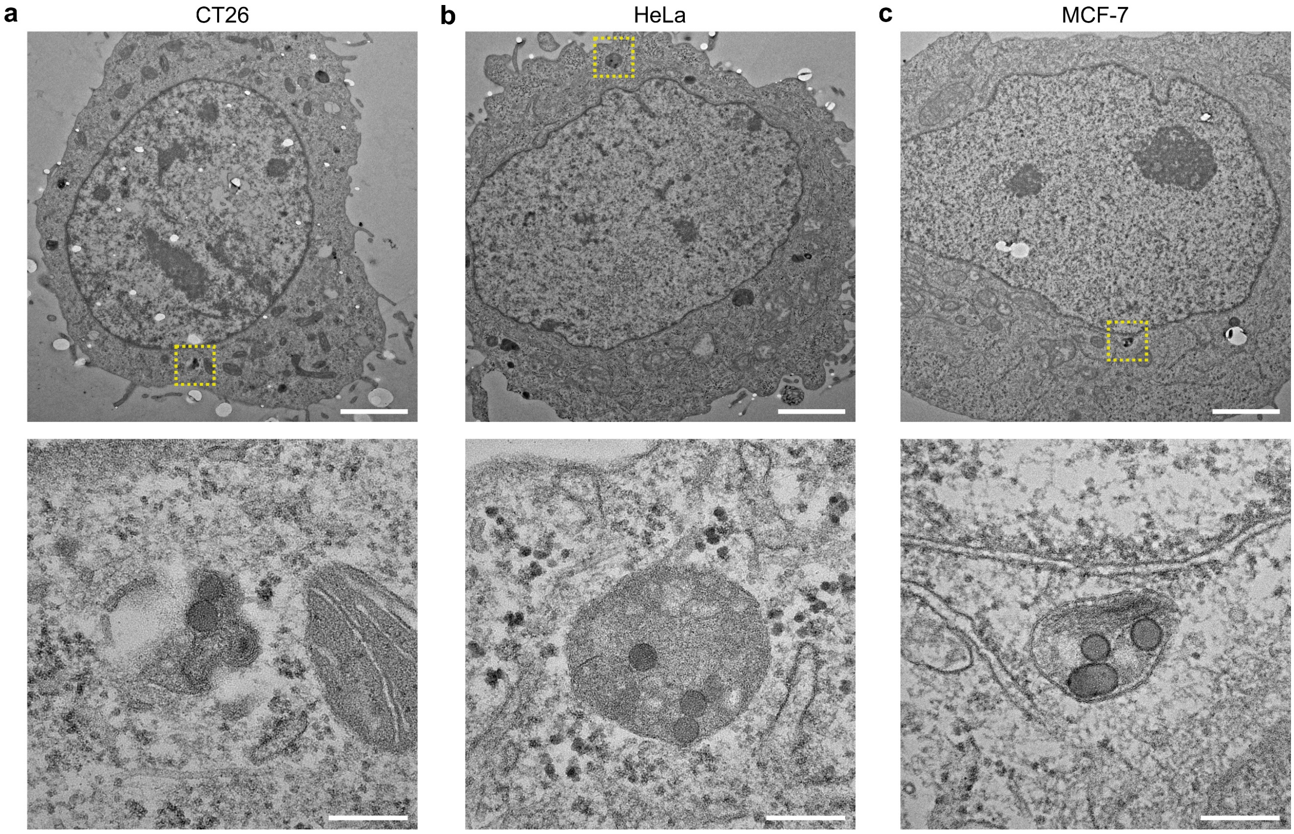

Supplementary Fig. 14 TEM images of CT26 cells (**a**), HeLa cells (**b**) and MCF-7 cells (**c**) after 4 h incubation with LB-SiO_2 NPs. Insets below are magnified of each image in the area highlighted with the respective yellow dashed box. Scale bars, 2 μm (top); 200 nm (bottom).

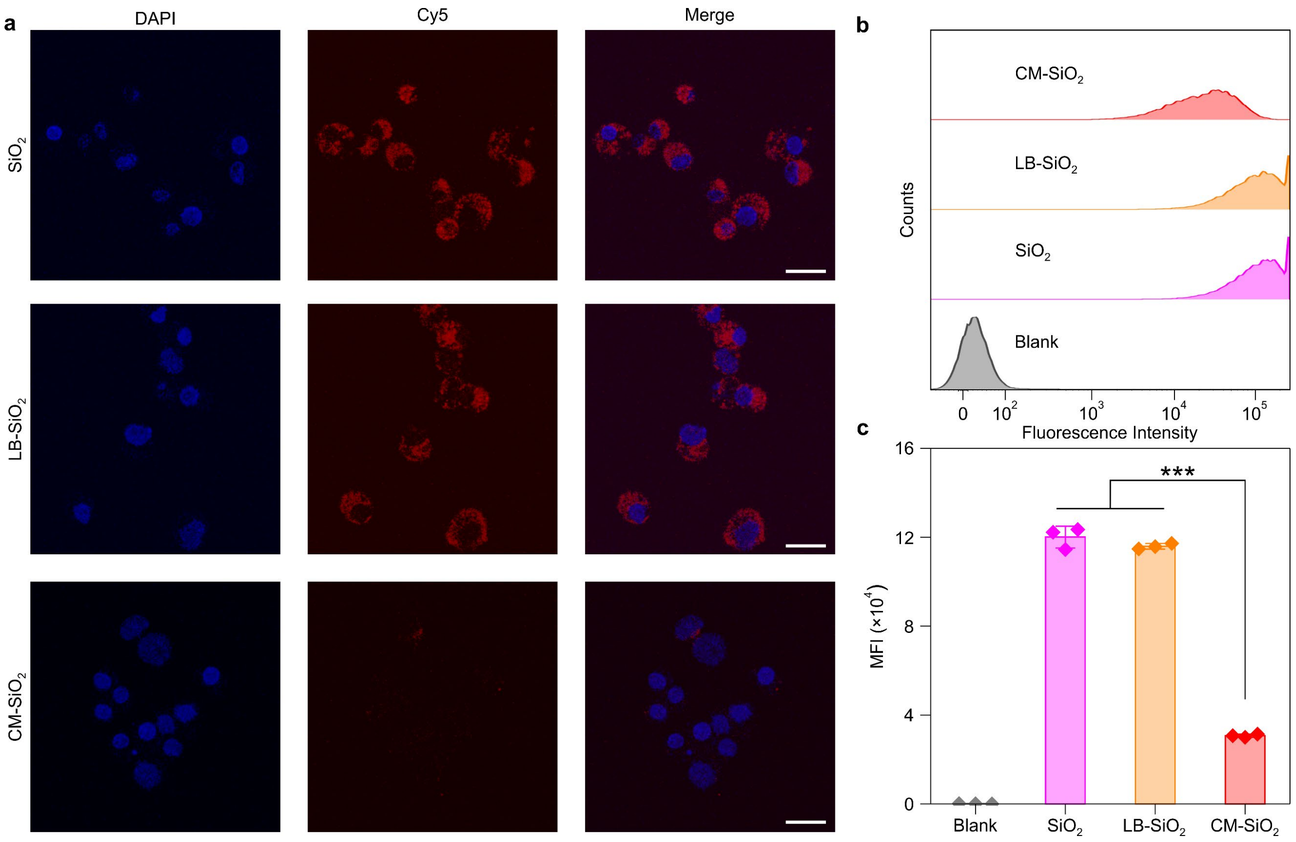

Supplementary Fig. 15 a, Representative CLSM images of RAW264.7 cells after 4 h incubation with SiO_2 NPs, LB-SiO_2 NPs and CM-SiO_2 NPs. The SiO_2 cores were labeled with Cy5 (red), and the cell nuclei were stained with DAPI (blue). Scale bars, 20 μm. **b,** Flow cytometric analysis of RAW264.7 cells incubated with blank solution, SiO_2 NPs, LB-SiO_2 NPs and CM-SiO_2 NPs. **c,** Quantification of cellular uptake in RAW264.7 cells. Data represents mean ± SD ($n = 3$). One-way ANOVA followed by post hoc Tukey test was used to determine the significance of data. ***$p < 0.001$.

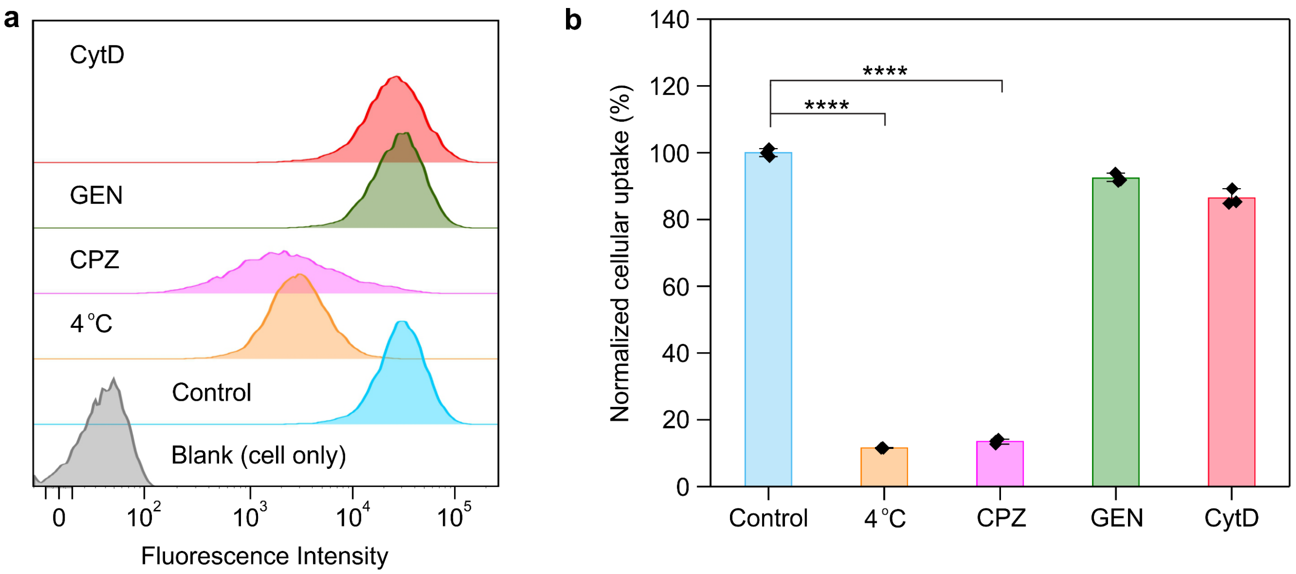

Supplementary Fig. 16 Influence of pharmacological inhibitors on CM-SiO_2 NPs uptake. Fluorescence intensity histograms (**a**) and fluorescence quantification (**b**) of cellular uptake by CM-SiO_2 NPs (50 μg/mL) in CT26 cells treated with low temperature (4 °C), chlorpromazine (CPZ), genistein (GEN), and cytochalasin D (CytD), as determined by flow cytometry. Data were normalized to cells treated only with CM-SiO_2 NPs at 37 °C in **b**. Data represents mean ± SD ($n = 3$). One-way ANOVA followed by post hoc Tukey test was used to determine the significance. ****$p < 0.0001$.

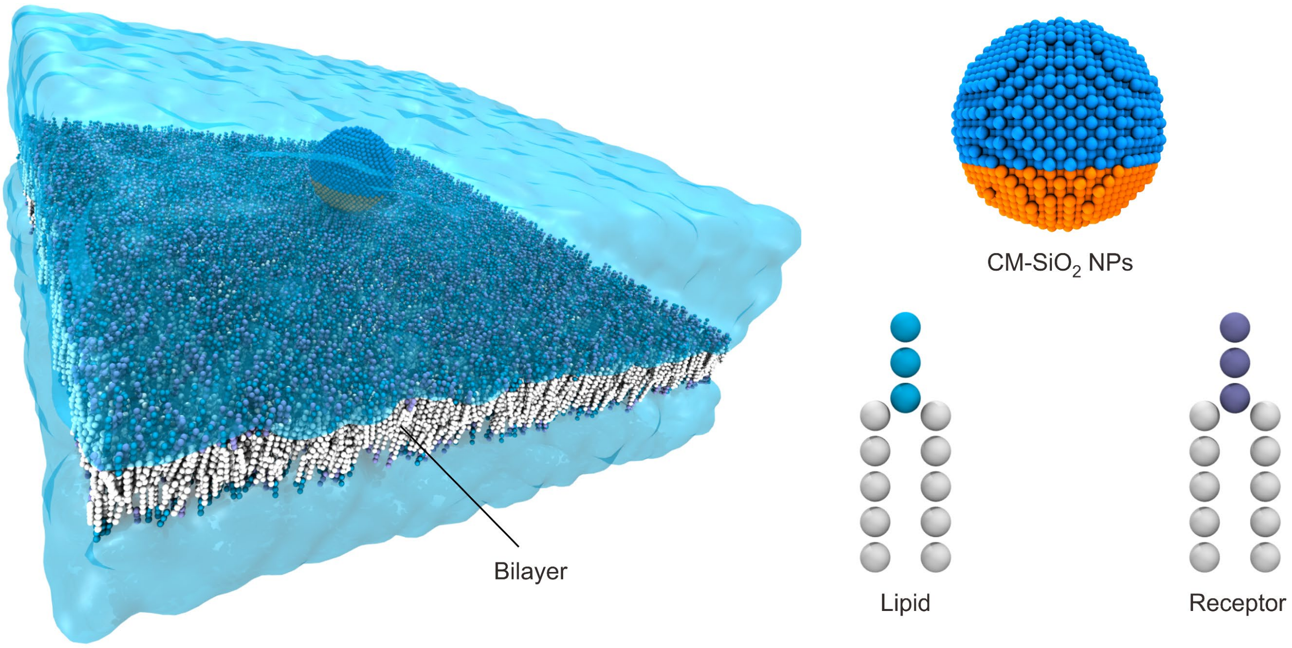

Supplementary Fig. 17 Schematic illustration of the models in the simulations. The coated part of the CM-SiO_2 NP is shown in orange and the uncoated part is shown in blue. For cell membranes, the heads and the tails of the membrane's phospholipids are shown in cyan and white, respectively. The receptor of the cellular membrane is shown in ice blue.

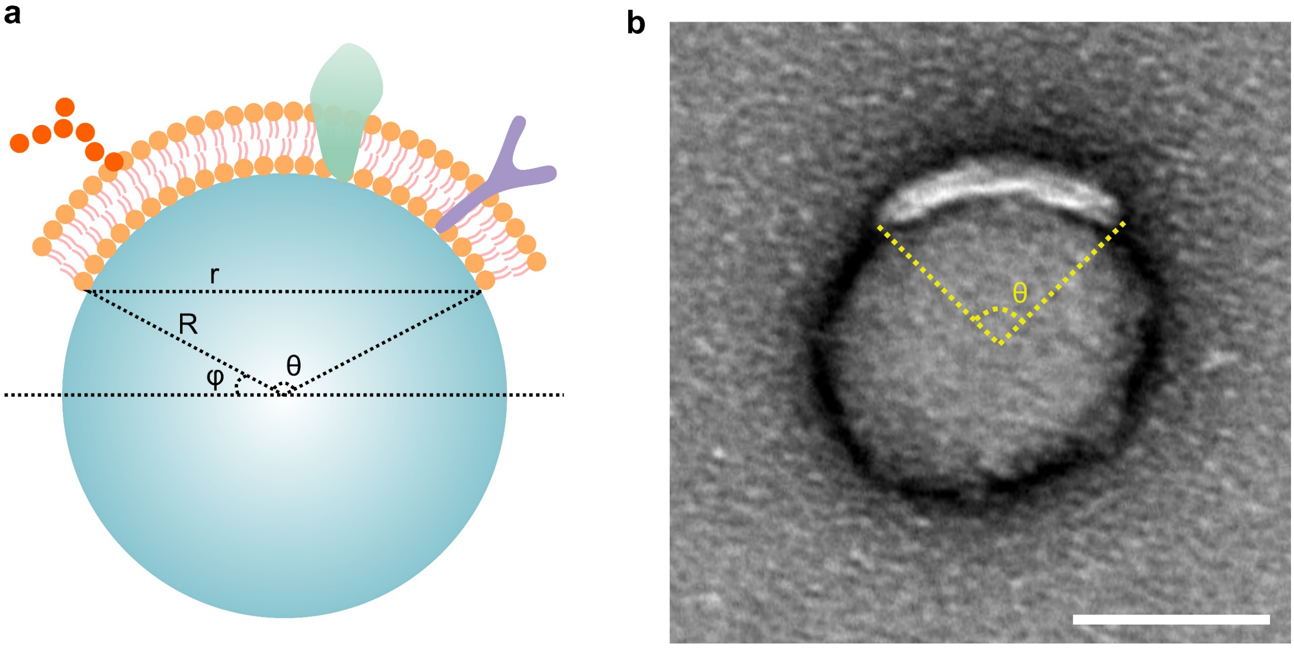

Supplementary Fig. 18 a, Schematic illustration of cell membrane coating degree calculation. **b,** TEM image of partially coated NPs. Scale bar, 50 nm. The calculation process is as follows:

The coating area of cell membrane:

$$S_{coating} = \int_0^{\frac{\pi}{2}} 2\pi rR \cdot d\varphi = \int_0^{\frac{\pi}{2}} 2\pi R^2 cos\varphi \cdot d\varphi = 2\pi R^2 \int_0^{\frac{\pi}{2}} cos\varphi \cdot d\varphi = 2\pi R^2(1 - sin\varphi),$$

Surface area of NPs: $S_{NPs} = 4\pi R^2$,

$$Coating\ degree(\%) = \frac{S_{coating}}{S_{NPs}} \times 100\% = \frac{2\pi R^2(1 - sin\varphi)}{4\pi R^2} \times 100\% = \frac{1 - sin\varphi}{2} \times 100\%$$
$$= \frac{1 - sin\left(\frac{\pi - \theta}{2}\right)}{2} \times 100\%,$$

Where θ can be measured directly from the TEM image (Supplementary Fig. 18b).

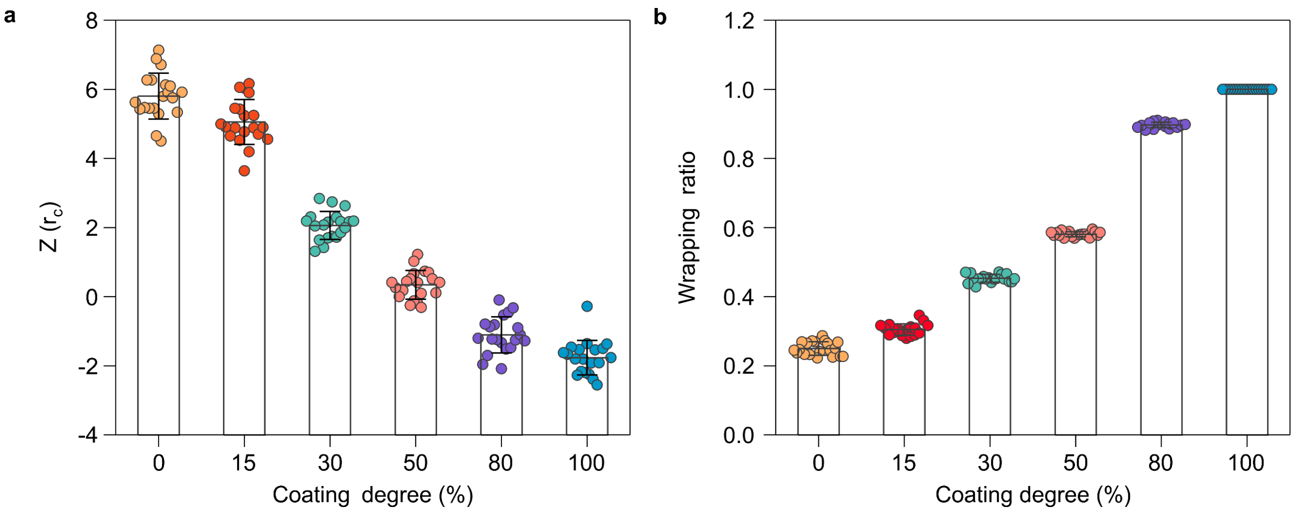

Supplementary Fig. 19 Effect of cell membrane coating degree on position (**a**) and wrapping ratio (**b**) of CM-SiO_2 NPs at the end of wrapping process. Data represents mean ± SD (*n* = 20).

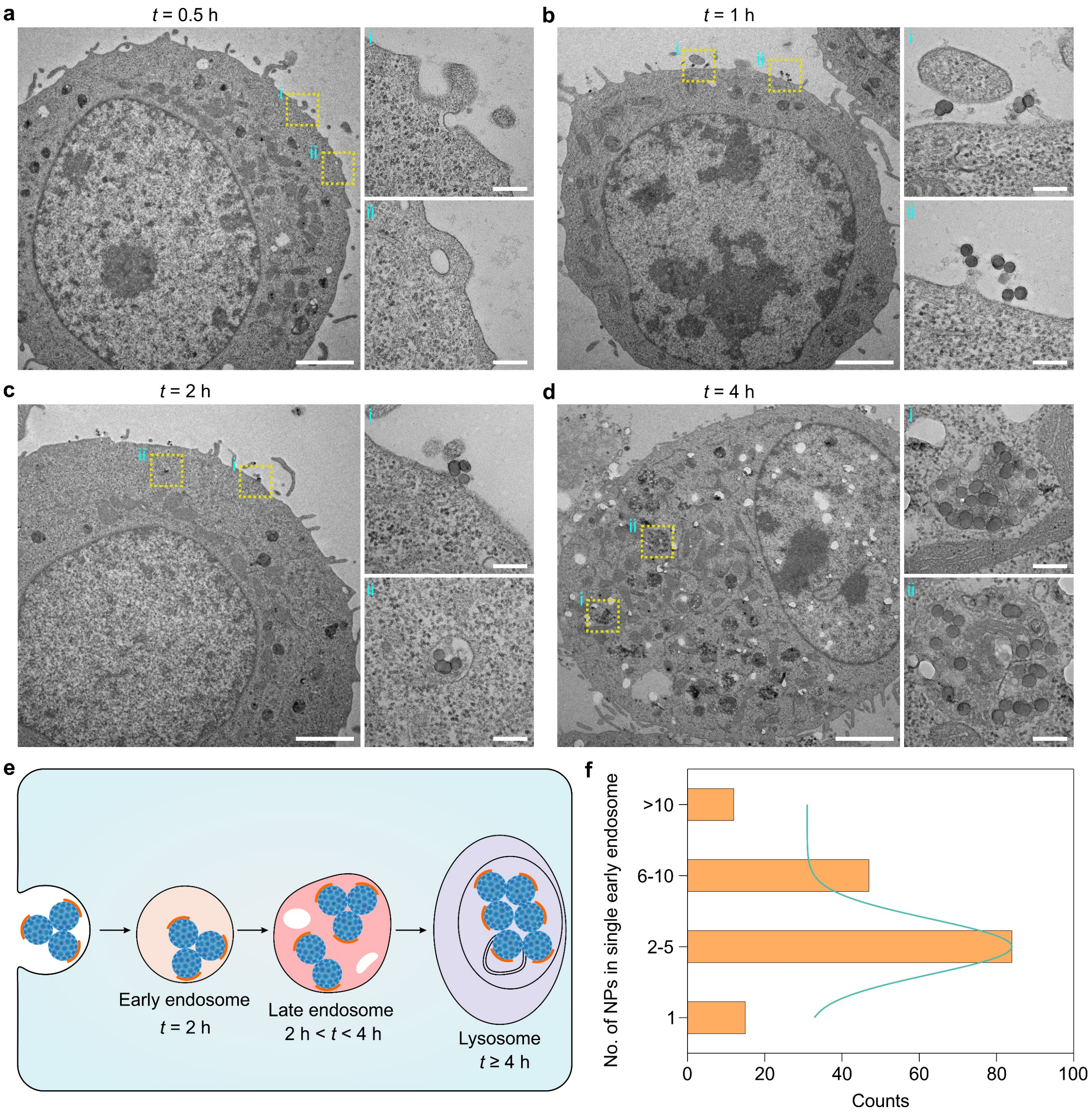

Supplementary Fig. 20 a-d, TEM images for time evolution of CM-SiO_2 NPs incubated with CT26 cells for 0.5 h (**a**), 1 h (**b**), 2 h (**c**), and 4 h (**d**). Scale bars, 2 μm; insets, 200 nm. **e,** Schematic of intracellular trafficking of CM-SiO_2 NPs. Partially coated NPs enter cells mainly in the multiple form and then those NPs are gradually trapped in early endosomes (2 h), late endosomes (2~4 h) and lysosomes (≥ 4 h) during the vesicle transport and maturation, respectively. **f,** Histogram distribution of the number of NPs trapped in a single early endosome. 158 early endosomes were analyzed from TEM images (2 h).

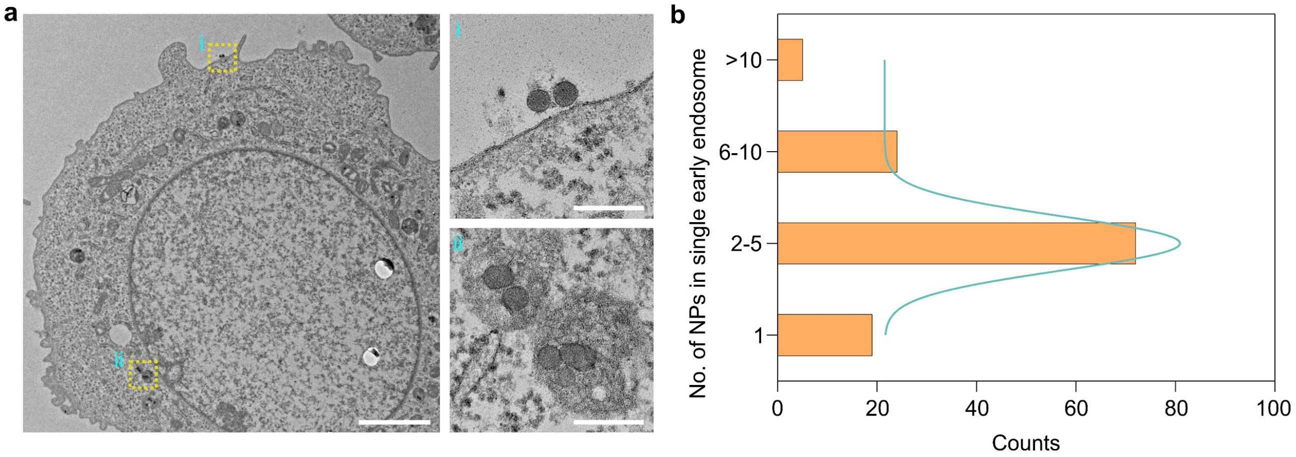

Supplementary Fig. 21 a, TEM images of CM-SiO_2 NPs (1 µg/mL) incubated with CT26 cells for 2 h. Scale bars, 2 µm; insets, 200 nm. **b,** Histogram distribution of the number of NPs trapped in a single early endosome. 120 early endosomes were analyzed from TEM images.

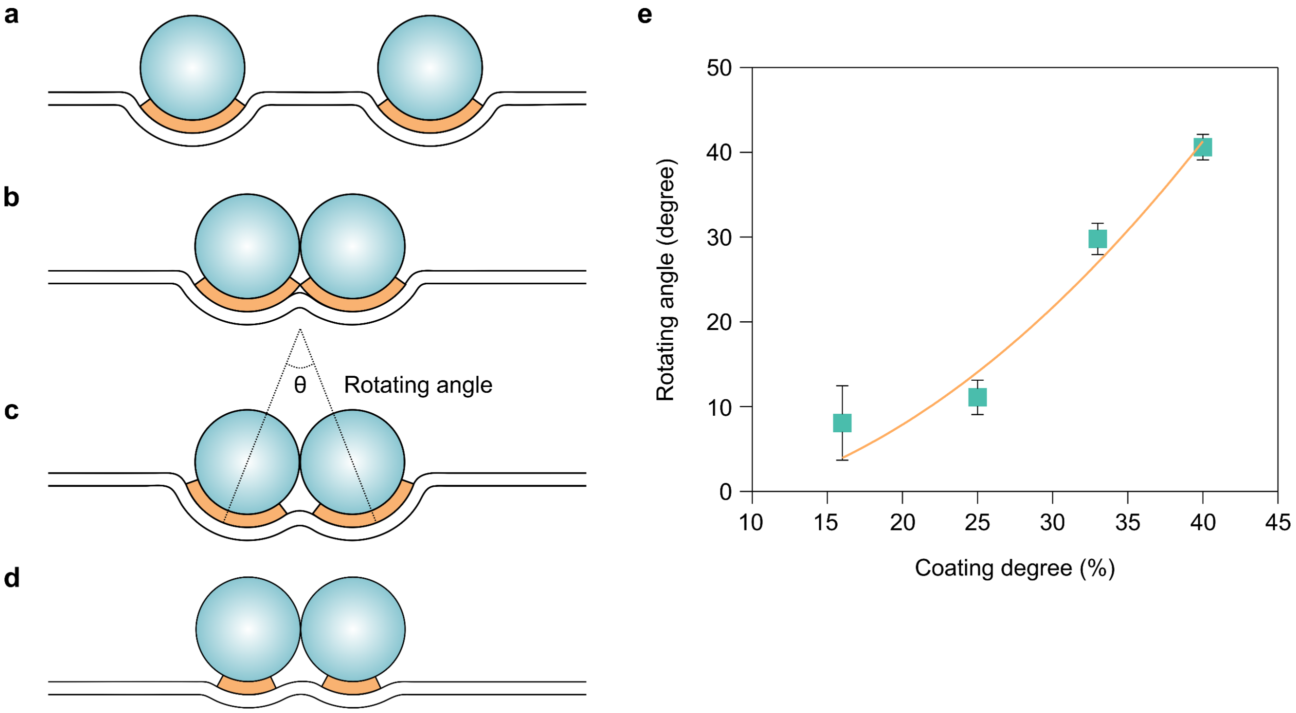

Supplementary Fig. 22 a-d, Schematic illustration of three different locations of two NPs when they interacted with the cell membrane: separated (**a**), contacted (**b**), and rotated with a high coating degree (30%; **c**) and a low coating degree (15%; **d**). **e,** Rotating angle as a function of coating degree. Data represents mean ± SD ($n = 20$).

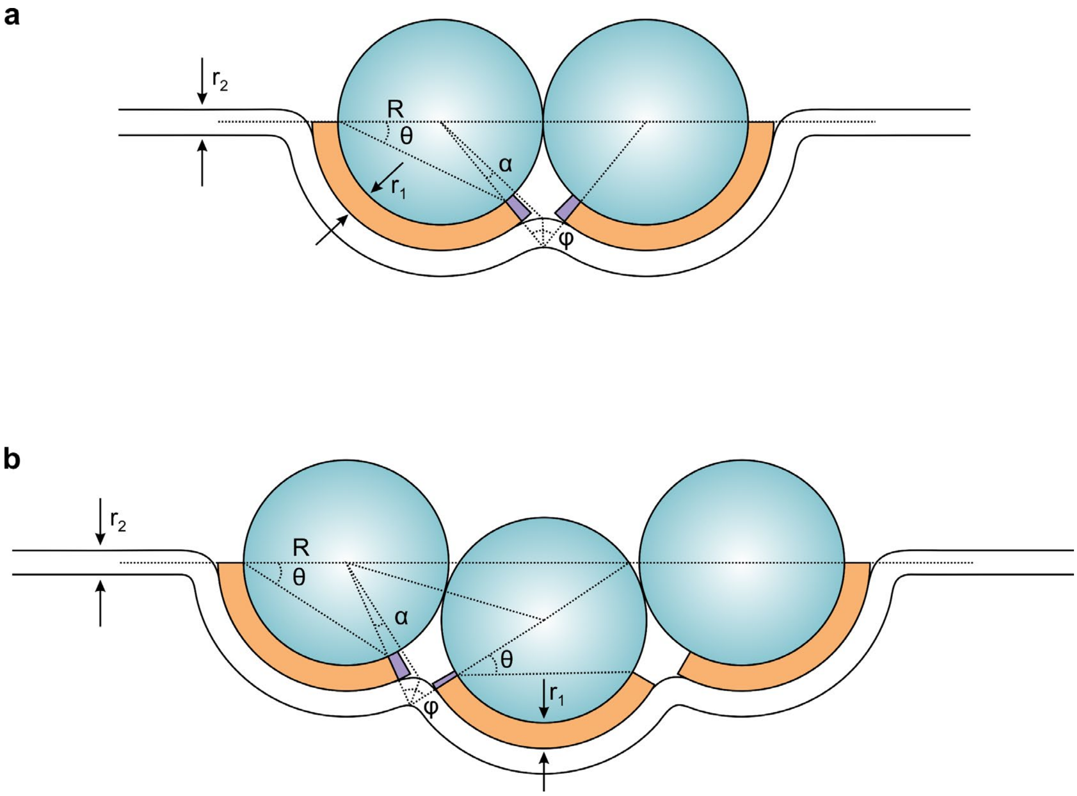

Supplementary Fig. 23 The two-dimension model applied to predict the required coating degree for half wrapping the NPs aggregates with n=2 (**a**) and n=3 (**b**). The orange region shows the coated region to drive the membrane bending around the CM-SiO_2 NPs. The purple region shows the coated region to drive the additional membrane deformation (including bending and stretching), which connects with the neighboring curved membrane. R, r_1 and r_2 stand for the radius of the SiO_2 NP, the thickness of the coating membrane and the thickness of the wrapping membrane, respectively.

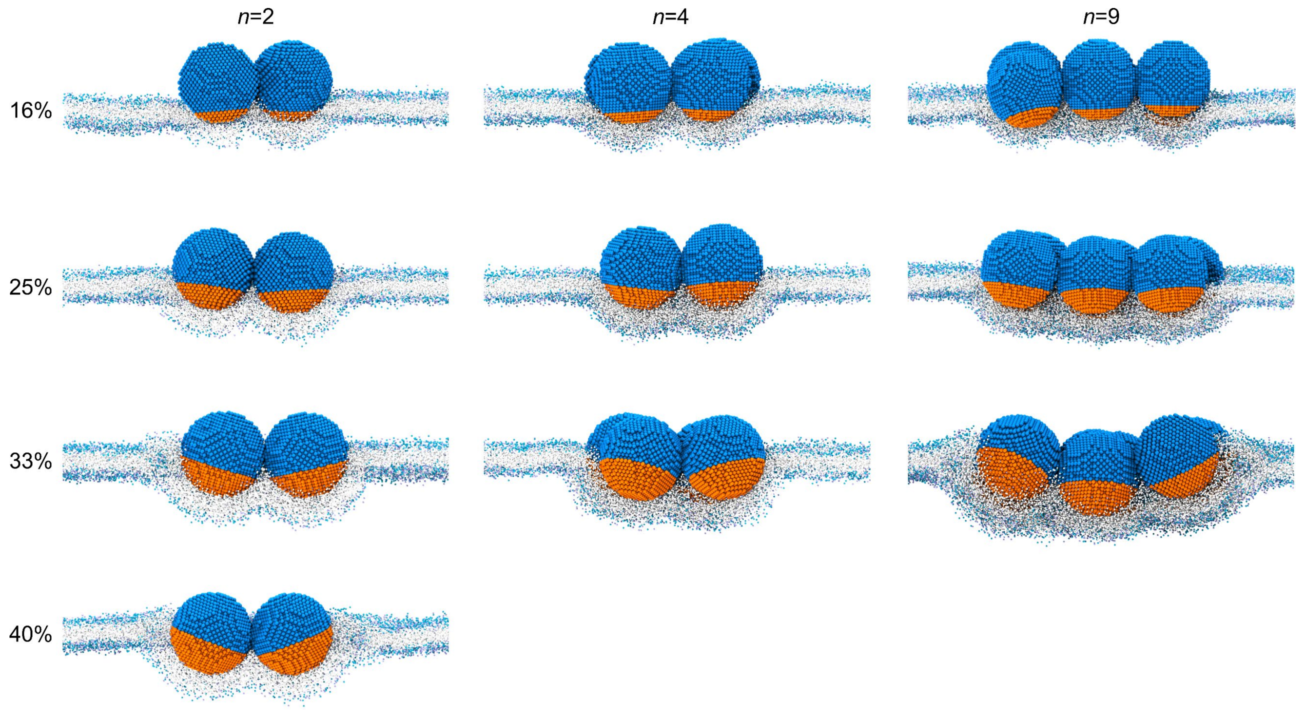

Supplementary Fig. 24 Final snapshots (t=160000 τ) of the interactions between different aggregated numbers (2, 4, and 9) of NPs and coating degrees (16%, 25%, 33% and 40%).

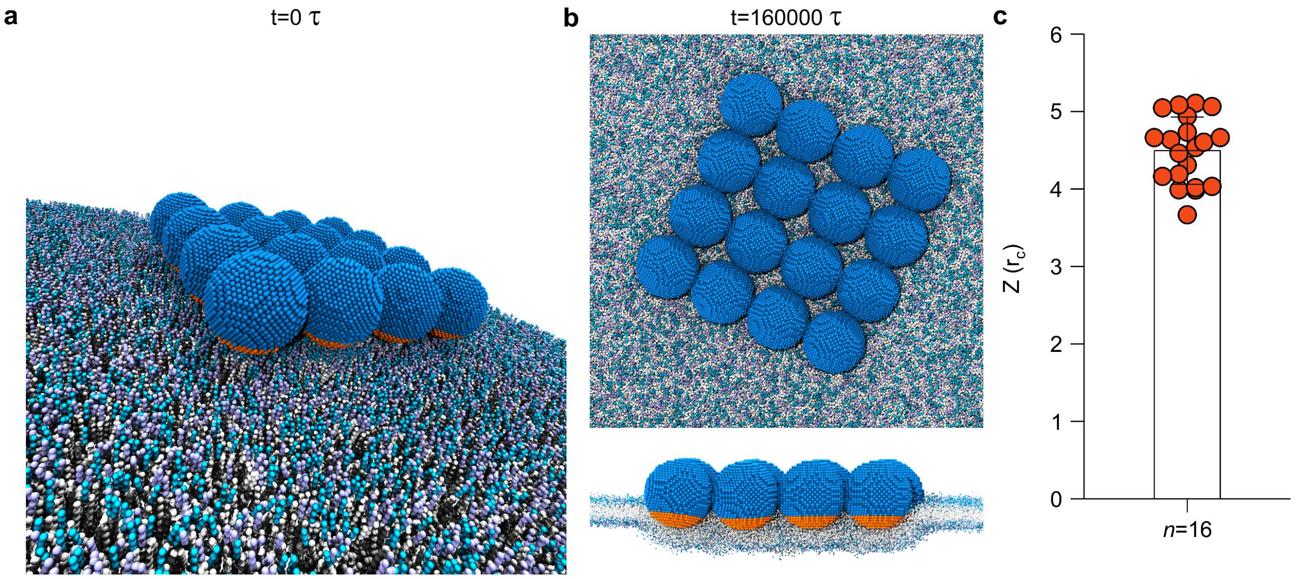

Supplementary Fig. 25 a,b, Representative DPD simulation snapshots of 16 aggregated CM-SiO_2 NPs show the setup of the simulation system (t=0 τ; **a**) and the final equilibrated NP-membrane structure at the top view and the profile view (t=160000 τ; **b**). The coating degree of each NP is 33%. **c,** Quantification of the final position of NPs. Data represents mean ± SD (*n* = 20).

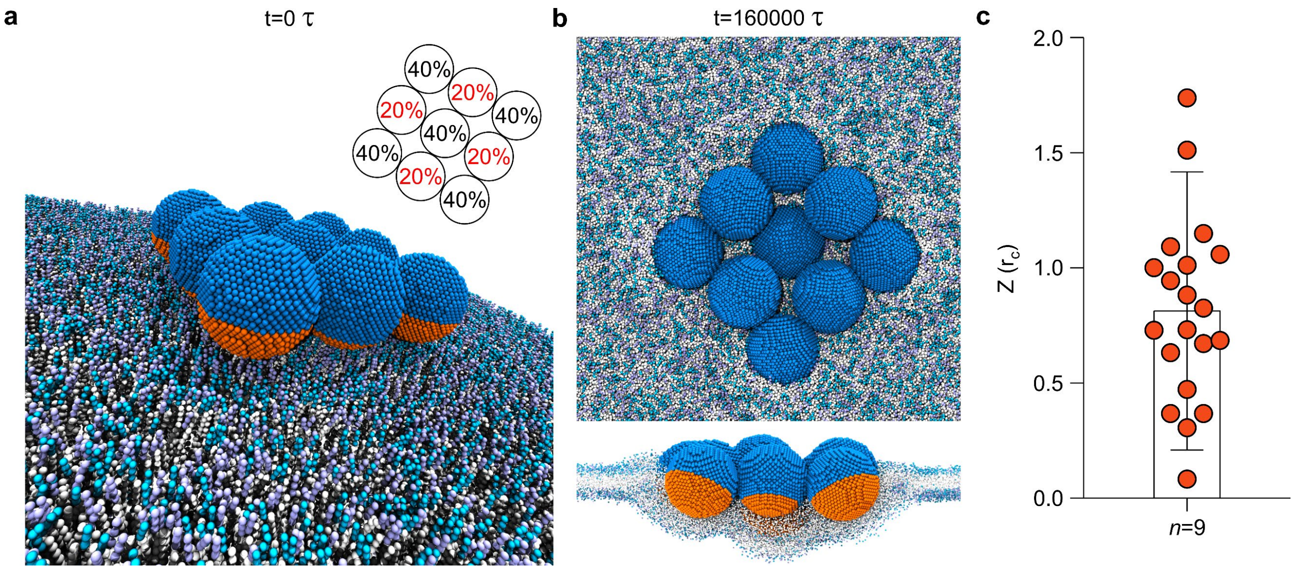

Supplementary Fig. 26 a,b, Representative DPD simulation snapshots of 9 aggregated CM-SiO_2 NPs with inconsistent coating degrees show the setup of the simulation system (t=0 τ; **a**) and the final equilibrated NP-membrane structure at the top view and the profile view (t=160000 τ; **b**). The distribution of the coating degree for each NP is shown in the inset. **c,** Quantification of the final position of NPs. Data represents mean ± SD (*n* = 20).

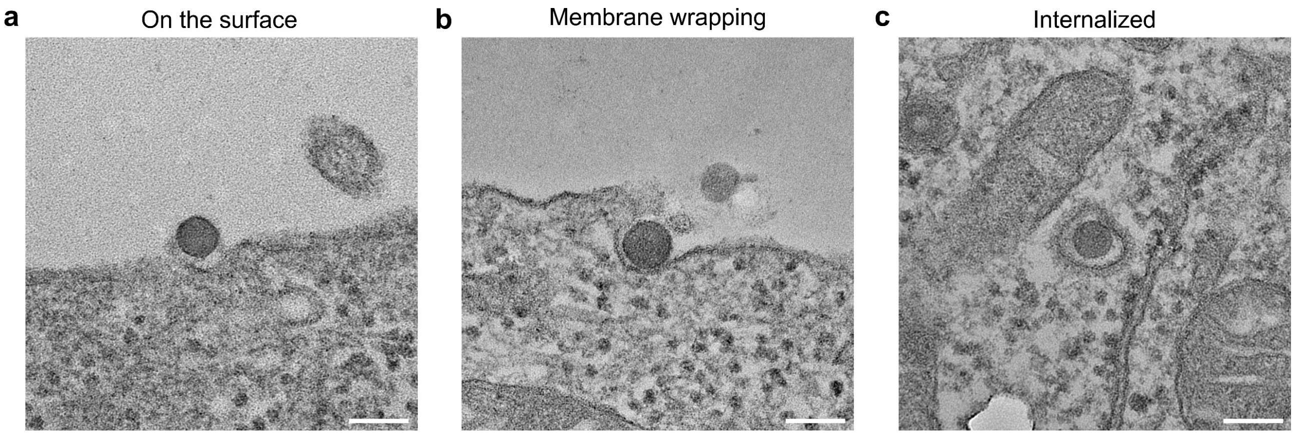

Supplementary Fig. 27 TEM images showing the process of a single CM-SiO_2 NP enter into CT26 cells. Scale bars, 100 nm.

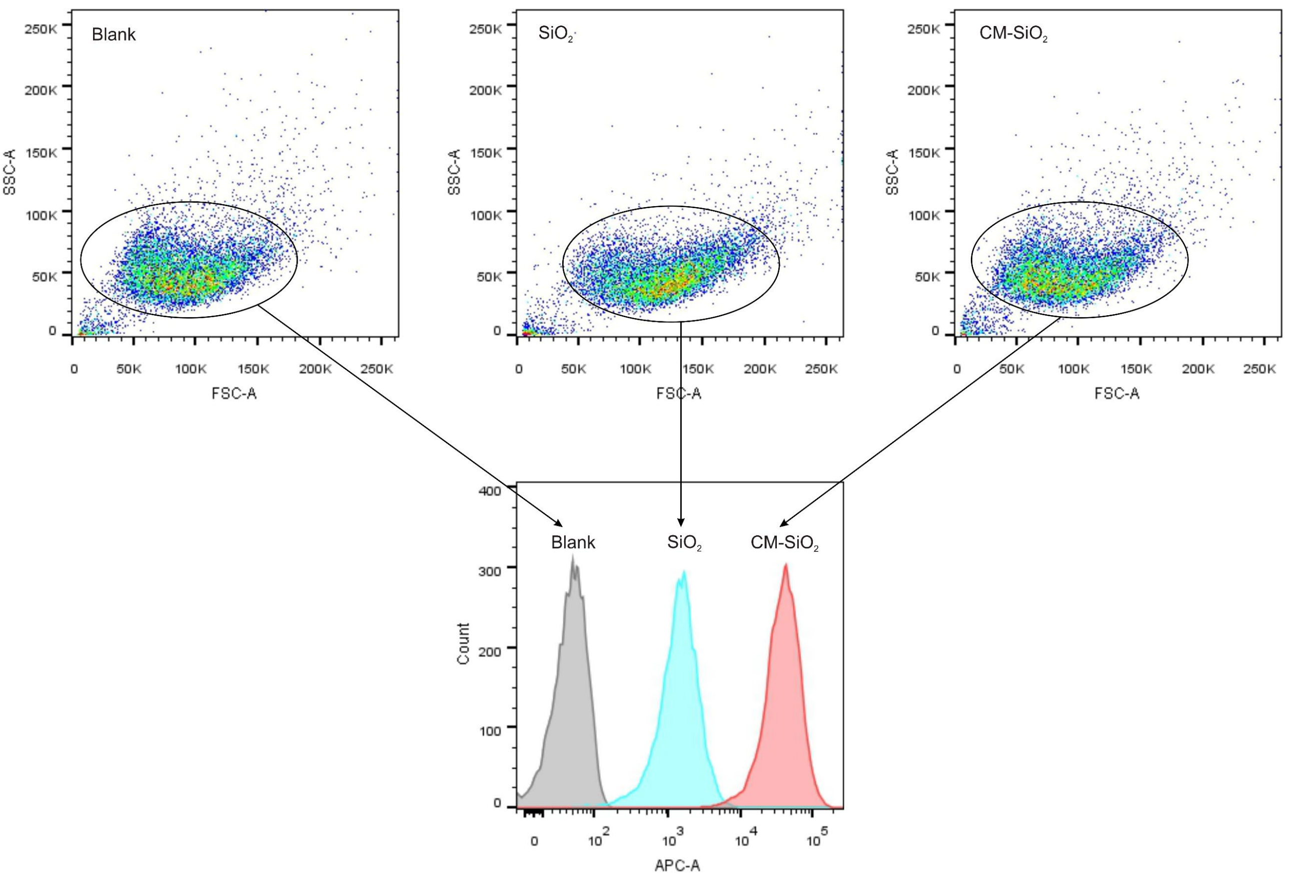

Supplementary Fig. 28 Gating strategy to quantify nanoparticle binding and uptake *in vitro* (Fig. 3b-d, Supplementary Fig. 15b and Supplementary Fig. 16a). Three times of this experiment was repeated independently with similar results.

Supplementary Tables

Supplementary Table 1 Synthesis conditions of nonporous Stöber SiO_2 NPs with a variety of sizes

Size (nm)	95% Ethanol (mL)	28-30% NH_4OH (mL)	TEOS (mL)	Temperature (K)
30 ± 2.9	40	0.6	0.5	298
62 ± 8.1	40	0.65	0.5	298
100 ± 7.0	40	1.1	0.5	298
149 ± 7.8	40	1.2	0.5	298
190 ± 8.6	40	1.5	0.5	298

Supplementary Table 2 Interaction parameters a_{ij} between the beads *i* and *j*. H, R, T and W represent the lipid head, receptor, lipid tail and water beads, respectively. P_1 and P_2 represent the coated and uncoated beads of the CM-SiO_2 NP, where the coated part is considered as the ligand and the uncoated part represents the SiO_2. The increase of a_{ij} represents the increase of the repulsion between the beads. Interaction parameters between the same beads a_{ii} are set as 25 except for P_2, which slightly increases to reflect the repulsion within the SiO_2 NPs by the surface charge coatings. The a_{ij} between the receptor and the ligand is set as 1 to mimic their strong interactions. Other parameters are described in the CG model section.

a_{ij}	H	R	T	W	P_1	P_2
H	25	25	100	25	25	25
R	25	25	100	25	1	25
T	100	100	25	100	100	100
W	25	25	100	25	25	40
P_1	25	1	100	25	25	25
P_2	25	25	100	40	25	30

Supplementary Movies

Supplementary Movies 1-3. Interaction between the cell membrane and the CM-SiO2 NPs with increasing the aggregation numbers: Supplementary Movie 1 (*n*=1), Supplementary Movie 2 (*n*=2), and Supplementary Movie 3 (*n*=9). The coating degree of each NP is 33%. It demonstrated that the higher number of NP aggregates were more likely to rotate spontaneously after entering into the membranes.

Supplementary Notes

Supplementary Note 1. Estimation of the coating degree by geometric constraints

With the geometric constraints and the symmetricity of the two-dimension model (Supplementary Fig. 23), the required coating degree (*c*) can be estimated by:

$$c_{\min} = \frac{A_{orange}}{A_{total}} = \frac{1-\sin\theta}{2} \tag{6}$$

$$c_{\max} = \frac{A_{orange} + A_{purple-max}}{A_{total}} = \frac{1-\sin\left(\theta-\frac{\alpha}{2}\right)}{2} \tag{7}$$

Here, the A_{orange} stands for the area of the orange region and $A_{purple-max}$ stands for the maximum of the area of the purple region. The θ and α can be derived by:

$$\theta = \frac{2\pi-\varphi}{4} \text{ (for 2 NPs)} \tag{8}$$

$$\theta = \frac{2\pi-\varphi}{3} \text{ (for 3 NPs)} \tag{9}$$

$$\alpha = \arctan\left(\frac{r_2 \sin\left(\frac{\varphi}{2}\right)}{R + r_1 + r_2 - r\cos\left(\frac{\varphi}{2}\right)}\right) \tag{10}$$

$$\varphi = 2\arcsin\left(\frac{R}{R + r_1 + r_2}\right) \tag{11}$$

According to the experimental conditions, the required coating degree for 2 NPs is: $c_{\min} = 29\%$ and $c_{\max} = 32\%$; the required coating degree for 3 NPs is: $c_{\min} = 23\%$ and $c_{\max} = 26\%$. According to the simulation conditions, the required coating degree for 2 NPs is: $c_{\min} = 29\%$ and $c_{\max} = 36\%$; the required coating degree for 3 NPs is: $c_{\min} = 23\%$ and $c_{\max} = 30\%$. When compared with the real three-dimension simulation results for 4 NPs (~33%) and 9 NPs (~30%), the estimated values are slightly larger. There are two possible reasons: 1. In the real three-dimension situation, the required coating degree for two dimensions needs to be satisfied, whereas only one dimension needs to be satisfied in a two-dimension model. 2. The actual additional deformation connecting the two neighboring curved membrane is much more complicated than the hypothetical one in the two-dimension model, which may need more driving energies provided by the coating membranes.

Supplementary References

1. Zou, M.Z. *et al.* A multifunctional biomimetic nanoplatform for relieving hypoxia to enhance chemotherapy and inhibit the PD-1/PD-L1 Axis. *Small* **14**, e1801120 (2018).
2. Deng, Y., Qi, D., Deng, C., Zhang, X. & Zhao, D. Superparamagnetic high-magnetization microspheres with an Fe_3O_4@ SiO_2 core and perpendicularly aligned mesoporous SiO_2 shell for removal of microcystins. *J. Am. Chem. Soc.* **130**, 28-29 (2008).
3. Hu, C.M. *et al.* Erythrocyte membrane-camouflaged polymeric nanoparticles as a biomimetic delivery platform. *Proc. Natl. Acad. Sci. USA* **108**, 10980-10985 (2011).
4. Nakki, S. *et al.* Improved stability and biocompatibility of nanostructured silicon drug carrier for intravenous administration. *Acta Biomater.* **13**, 207-215 (2015).
5. Kim, S.H., Jeong, J.H., Chun, K.W. & Park, T.G. Target-specific cellular uptake of PLGA nanoparticles coated with poly (l-lysine)−poly (ethylene glycol)−folate conjugate. *Langmuir* **21**, 8852-8857 (2005).
6. Hanawa-Suetsugu, K. *et al.* Phagocytosis is mediated by two-dimensional assemblies of the F-BAR protein GAS7. *Nat. Commun.* **10**, 4763 (2019).
7. Liu, J., Stace-Naughton, A., Jiang, X. & Brinker, C.J. Porous nanoparticle supported lipid bilayers (protocells) as delivery vehicles. *J. Am. Chem. Soc.* **131**, 1354-1355 (2009).
8. Yang, K. & Ma, Y.Q. Computer simulation of the translocation of nanoparticles with different shapes across a lipid bilayer. *Nat. Nanotechnol.* **5**, 579-583 (2010).
9. Ding, H.m. & Ma, Y.q. Theoretical and computational investigations of nanoparticle–biomembrane interactions in cellular delivery. *Small* **11**, 1055-1071 (2015).
10. Alexeev, A., Uspal, W.E. & Balazs, A.C. Harnessing Janus nanoparticles to create controllable pores in membranes. *ACS Nano* **2**, 1117-1122 (2008).
11. Hoogerbrugge, P. & Koelman, J. Simulating microscopic hydrodynamic phenomena with dissipative particle dynamics. *Europhys. Lett.* **19**, 155-160 (1992).
12. Groot, R.D. & Warren, P.B. Dissipative particle dynamics: Bridging the gap between atomistic and mesoscopic simulation. *J. Chem. Phys.* **107**, 4423-4435 (1997).
13. Plimpton, S. Fast parallel algorithms for short-range molecular dynamics. *J. Comput. Phys.* **117**, 1-19 (1995).
