# Supplementary material for: Cell membrane coating integrity affects the internalization mechanism of biomimetic nanoparticles
Source: Nat Commun. 2021 Sep 30;12:5726. doi: 10.1038/s41467-021-26052-x (PMC8484581; doi:10.1038/s41467-021-26052-x)
Supplement: Supplementary file 2 — Description of Additional Supplementary Files [file 41467_2021_26052_MOESM2_ESM.docx]

**Description of Additional Supplementary Files:**

**Supplementary Movies 1-3:**

Interaction between the cell membrane and the CM-SiO2 NPs with increasing the aggregation numbers:

**Supplementary Movie 1 (n=1)**

**Supplementary Movie 2 (n=2)**

**Supplementary Movie 3 (n=9).**

The coating degree of each NP is 33%. It demonstrated that the higher number of NP aggregates were more likely to rotate spontaneously after entering into the membranes.
