# Supplementary material for: Cell membrane coating integrity affects the internalization mechanism of biomimetic nanoparticles
Source: Nat Commun. 2021 Sep 30;12:5726. doi: 10.1038/s41467-021-26052-x (PMC8484581; doi:10.1038/s41467-021-26052-x)
Supplement: Supplementary file 6 — Reporting Summary [file 41467_2021_26052_MOESM6_ESM.pdf]

nature portfolio

Corresponding author(s): Vesa-Pekka Lehto, Wujun Xu

Last updated by author(s): Aug 20, 2021

Reporting Summary

Nature Portfolio wishes to improve the reproducibility of the work that we publish. This form provides structure for consistency and transparency in reporting. For further information on Nature Portfolio policies, see our Editorial Policies and the Editorial Policy Checklist.

Statistics

For all statistical analyses, confirm that the following items are present in the figure legend, table legend, main text, or Methods section.

n/a	Confirmed	
☐	☒	The exact sample size (*n*) for each experimental group/condition, given as a discrete number and unit of measurement
☐	☒	A statement on whether measurements were taken from distinct samples or whether the same sample was measured repeatedly
☐	☒	The statistical test(s) used AND whether they are one- or two-sided<br>*Only common tests should be described solely by name; describe more complex techniques in the Methods section.*
☒	☐	A description of all covariates tested
☒	☐	A description of any assumptions or corrections, such as tests of normality and adjustment for multiple comparisons
☐	☒	A full description of the statistical parameters including central tendency (e.g. means) or other basic estimates (e.g. regression coefficient) AND variation (e.g. standard deviation) or associated estimates of uncertainty (e.g. confidence intervals)
☐	☒	For null hypothesis testing, the test statistic (e.g. *F*, *t*, *r*) with confidence intervals, effect sizes, degrees of freedom and *P* value noted<br>*Give P values as exact values whenever suitable.*
☒	☐	For Bayesian analysis, information on the choice of priors and Markov chain Monte Carlo settings
☒	☐	For hierarchical and complex designs, identification of the appropriate level for tests and full reporting of outcomes
☒	☐	Estimates of effect sizes (e.g. Cohen's *d*, Pearson's *r*), indicating how they were calculated

Our web collection on statistics for biologists contains articles on many of the points above.

Software and code

Policy information about availability of computer code

Data collection	FlowJo V10, ZEN 3.1 (blue edition), Malvern Zetasizer Nano ZS, ImageJ, OMNIC 8.2, LAMMPS package.
Data analysis	Flow cytometry results were analyzed by FlowJo V10. Statistical calculations were performed using Origin 2019 software (OriginLabs).

For manuscripts utilizing custom algorithms or software that are central to the research but not yet described in published literature, software must be made available to editors and reviewers. We strongly encourage code deposition in a community repository (e.g. GitHub). See the Nature Portfolio guidelines for submitting code & software for further information.

Data

Policy information about availability of data

All manuscripts must include a data availability statement. This statement should provide the following information, where applicable:
- Accession codes, unique identifiers, or web links for publicly available datasets
- A description of any restrictions on data availability
- For clinical datasets or third party data, please ensure that the statement adheres to our policy

The authors declare that all data supporting the findings of this study are available within the paper and its supplementary information files or available from the corresponding author upon reasonable request.

Field-specific reporting

Please select the one below that is the best fit for your research. If you are not sure, read the appropriate sections before making your selection.

- [x] Life sciences
- [ ] Behavioural & social sciences
- [ ] Ecological, evolutionary & environmental sciences

For a reference copy of the document with all sections, see nature.com/documents/nr-reporting-summary-flat.pdf

Life sciences study design

All studies must disclose on these points even when the disclosure is negative.

Sample size	Samples sizes were determined based on previous experience and by referencing previously published studies.
Data exclusions	No data was excluded in this study.
Replication	At least three independent samples were performed for each experiment. All experiments were performed as technical or biological replications as appropriate for the experimental design.
Randomization	All samples were randomly allocated into experimental groups.
Blinding	Investigators were blinded to group allocation during data collection and analysis.

Reporting for specific materials, systems and methods

We require information from authors about some types of materials, experimental systems and methods used in many studies. Here, indicate whether each material, system or method listed is relevant to your study. If you are not sure if a list item applies to your research, read the appropriate section before selecting a response.

Materials & experimental systems

n/a	Involved in the study	
x		Antibodies
	x	Eukaryotic cell lines
x		Palaeontology and archaeology
x		Animals and other organisms
x		Human research participants
x		Clinical data
x		Dual use research of concern

Methods

n/a	Involved in the study	
x		ChIP-seq
	x	Flow cytometry
x		MRI-based neuroimaging

Eukaryotic cell lines

Policy information about cell lines

Cell line source(s)	The cell lines used in this study are CT26, HeLa, MCF-7 and macrophage RAW264.7, which were purchased from the American Type Culture Collection (ATCC).
Authentication	The cell lines used in the study were no authenticated.
Mycoplasma contamination	All the cell lines were tested negative for mycoplasma contamination.
Commonly misidentified lines (See ICLAC register)	None

Flow Cytometry

Plots

Confirm that:

- [x] The axis labels state the marker and fluorochrome used (e.g. CD4-FITC).
- [x] The axis scales are clearly visible. Include numbers along axes only for bottom left plot of group (a 'group' is an analysis of identical markers).
- [ ] All plots are contour plots with outliers or pseudocolor plots.
- [x] A numerical value for number of cells or percentage (with statistics) is provided.

Methodology

Sample preparation	In the flow cytometric analysis, Cy5 labelled SiO2 NPs were used to track the uptake of the CM-SiO2 NPs. Initially, different cell lines were seeded in 6-well plates at a density of 5×105 cells per single well and cultured for 24 h in 2 mL of DMEM or RPMI 1640 medium containing 10 % FBS and 1% antibiotic antimycotic solution (100×). After SiO2 NPs, LB-SiO2 NPs and CM-SiO2 NPs (50 μg/mL) were coincubated with the cells for 4 h, then the cells were washed three times with HBSS, detached by trypsin-EDTA and finally collected by centrifugation at 1200×g for 5 min. The bottom cells were washed three times with HBSS and then the suspended cells were analyzed by flow cytometry.
Instrument	BD FACSCanto™ II (BD Biosciences, San Jose, CA USA)
Software	FlowJo V10
Cell population abundance	No cell sorting was performed.
Gating strategy	The preliminary FSC/SSC gates were determined by the blank cell samples.

☒ Tick this box to confirm that a figure exemplifying the gating strategy is provided in the Supplementary Information.
